# A human endogenous retrovirus encoded protease potentially cleaves numerous cellular proteins

**DOI:** 10.1186/s13100-019-0178-z

**Published:** 2019-08-22

**Authors:** Giuseppe Rigogliuso, Martin L. Biniossek, John L. Goodier, Bettina Mayer, Gavin C. Pereira, Oliver Schilling, Eckart Meese, Jens Mayer

**Affiliations:** 1 Department of Human Genetics, Medical Faculty, University of Saarland, Homburg, Germany; 2 Institute of Molecular Medicine and Cell Research, University of Freiburg, Freiburg, Germany; 3 McKusick-Nathans Institute of Genetic Medicine, Johns Hopkins University School of Medicine, Baltimore, MD USA; 4 Institute of Surgical Pathology, Medical Center, University of Freiburg, Freiburg, Germany; 5 German Cancer Consortium (DKTK) and German Cancer Research Center (DKFZ), Heidelberg, Germany

**Keywords:** HERV-K, Endogenous retrovirus, Retroviral protease, Proteolysis, Pathogenesis, Retrotransposon

## Abstract

**Background:**

A considerable portion of the human genome derives from retroviruses inherited over millions of years. Human endogenous retroviruses (HERVs) are usually severely mutated, yet some coding-competent HERVs exist. The HERV-K(HML-2) group includes evolutionarily young proviruses that encode typical retroviral proteins. HERV-K(HML-2) has been implicated in various human diseases because transcription is often upregulated and some of its encoded proteins are known to affect cell biology. HERV-K(HML-2) Protease (Pro) has received little attention so far, although it is expressed in some disease contexts and other retroviral proteases are known to process cellular proteins.

**Results:**

We set out to identify human cellular proteins that are substrates of HERV-K(HML-2) Pro employing a modified Terminal Amine Isotopic Labeling of Substrates (TAILS) procedure. Thousands of human proteins were identified by this assay as significantly processed by HERV-K(HML-2) Pro at both acidic and neutral pH. We confirmed cleavage of a majority of selected human proteins in vitro and in co-expression experiments in vivo. Sizes of processing products observed for some of the tested proteins coincided with product sizes predicted by TAILS. Processed proteins locate to various cellular compartments and participate in diverse, often disease-relevant cellular processes. A limited number of HERV-K(HML-2) reference and non-reference loci appears capable of encoding active Pro.

**Conclusions:**

Our findings from an approach combining TAILS with experimental verification of candidate proteins in vitro and in cultured cells suggest that hundreds of cellular proteins are potential substrates of HERV-K(HML-2) Pro. It is therefore conceivable that even low-level expression of HERV-K(HML-2) Pro affects levels of a diverse array of proteins and thus has a functional impact on cell biology and possible relevance for human diseases. Further studies are indicated to elucidate effects of HERV-K(HML-2) Pro expression regarding human substrate proteins, cell biology, and disease. The latter also calls for studies on expression of specific HERV-K(HML-2) loci capable of encoding active Pro. Endogenous retrovirus-encoded Pro activity may also be relevant for disease development in species other than human.

**Electronic supplementary material:**

The online version of this article (10.1186/s13100-019-0178-z) contains supplementary material, which is available to authorized users.

## Background

Human endogenous retroviruses (HERVs), originating from past infections by exogenous retroviruses, and derived elements with some retroviral features, comprise about 8% of the human genome. HERVs affect the biology of the human genome in various ways, ranging from influences on transcription and splicing to biological effects of retrovirus-like proteins still encoded by some HERV groups. For instance, the envelope glycoprotein encoded by a provirus of the so-called HERV-W group was coopted to form the *ERVW-1 (Syncytin-1)* gene, whose protein product exerts important functions in human placenta development and functionality [1]. The HERV-K(HML-2) group, in short HML-2, includes a number of evolutionarily young proviruses, several of which are human-specific or even polymorphic in the human population [2]. Transcription of some HML-2 loci is upregulated in various human diseases with potential consequences due to the interaction of HML-2-encoded proteins with other cellular proteins (for reviews, see [3–5]). For instance, certain types of testicular and ovarian germ cell tumors (GCTs), as well as melanoma and mammary carcinomas, display upregulated HML-2 transcription (reviewed in [6, 7]). Upregulated HML-2 transcription could be observed in lesions considered precursors of testicular GCTs, so-called carcinoma in situ of the testis [8]. GCT patients suffering from GCT-types with HML-2 upregulation already show a strong humoral response against HML-2-encoded Gag and Env proteins at the time of tumor detection [9, 10]. HML-2 encoded Env protein was recently shown to induce several transcription factors and to activate the cellular transformation-associated MAPK ERK1/2 pathway [11]. HML-2 Rec and Np9 proteins, encoded by spliced transcripts from the HML-2 *env* gene, were shown to interact with several human proteins, among them promyelocytic zinc finger protein (PLZF), testicular zinc finger protein (TZFP), Staufen-1, human small glutamine-rich (hSGT), and ligand of Numb protein X (LNX). Rec expression disturbed germ cell development in mice and altered testis histology towards a carcinoma-like phenotype [12–18].

Retroviral genomes usually encode several catalytic proteins, among them aspartyl Protease (Pro). HML-2 also encodes Pro that, after self-processing from a Gag-Pro(−Pol) precursor translated through ribosomal frameshifts, cleaves retroviral HML-2 Gag protein into matrix, capsid and nucleocapsid domains, as is typical for other retroviral aspartyl proteases [19–21]. There is strong evidence that active HML-2 Pro is expressed at significant amounts and during longer periods of time, especially for GCT. HML-2-encoded retroviral particles budding from GCT cell lines have been detected. Large amounts of HML-2 Gag protein are present in GCT tissue and HML-2 Pro-cleaved Gag protein was demonstrated in GCT cell lines and especially tissue samples [10, 22]. Bieda et al. [23] demonstrated mature HML-2-encoded retroviral particles budding from different GCT cell lines, immature non-budding retroviral particles, as well as cleaved Gag protein in those cell lines. Prokaryotic expression of a construct harboring HML-2 Gag-Pro ORFs results in self-processing of Pro from a Gag-Pro precursor [24], thus Pro is capable of self-processing independent of retroviral particle formation and budding.

Besides retroviral Gag protein, retroviral aspartyl proteases were found to cleave host cellular proteins. HIV Pro processes human Actin, Troponin C, Alzheimer amyloid precursor protein, and Pro-interleukin 1β in vivo. Purified HIV Pro processes Vimentin, Desmosin, and Glial fibrillary acidic protein, and Microtuble-associated proteins 1 and 2 in vitro (reviewed in [25]). Riviere et al. [26] reported processing of the precursor of NF-kappa B by HIV-1 Pro during acute infection. Processing of Vimentin by proteases of Bovine Leukemia Virus, Mason–Pfizer Monkey Virus, and Myeloblastosis-Associated Virus was reported by Snásel et al. [27]. Shoeman et al. [28] reported cleavage of focal adhesion plaque proteins, including Fimbrin, Focal adhesion plaque kinase, Talin, Filamin, Spectrin and Fibronectin by HIV-1 and HIV-2 proteases. Devroe at al. [29] reported processing of human NDR1 and NDR2 serine-threonine kinases by HIV-1 Pro. More recently, more than 120 cellular substrates were reported to be processed by HIV-1 Pro in vitro by Impens et al. [30]. Thus, aspartyl proteases from diverse retroviruses appear able to degrade quite a number of host cellular proteins. Furthermore, such processing of cellular proteins by retroviral Pro can occur independent of retroviral budding. For instance, cleavage of procaspase 8 by HIV-1 Pro was observed during HIV-1 infection of T-cells and other cell types [31, 32]. HIV-1 Pro was reported to cleave serine-threonine kinases RIPK1 and RIPK2 during HIV-1 infection of T-cell lines or primary activated CD4^+^ T cells ([33], see references therein for additional examples). A significant amount of processing of HIV-1 Gag occurs in the cytoplasm of infected cells resulting in intracellular accumulation of appropriately processed HIV-1 Gag proteins [34]. For Mouse Mammary Tumor Virus (MMTV), a betaretrovirus closely related with HERV-K(HML-2), activation of Pro can occur before budding, and MMTV Gag protein is primarily found in the cytoplasm and traffics to intracellular membranes to initiate particle assembly. Similar observations were made for Human Foamy Virus [35–37]. Thus, retroviral Pro proteins are activated not only during maturation of retroviral particles.

There is evidence that such processing of cellular proteins by retroviral Pro is of biological relevance. Strack et al. [38] reported that apoptosis of HIV-infected cells was preceded by HIV Pro-mediated cleavage of Bcl-2. Cleavage of Procaspase 8 by HIV Pro in T-cells was followed by cellular events characteristic of apoptosis [31]. HIV Pro inducibly expressed in yeast caused cell lysis due to alterations in membrane permeability. Cell killing and lysis, specifically lysis by necrosis without signs of apoptosis, was observed in COS-7 cells following expression of HIV Pro [39]. Cleavage of EIF4G by several retroviral proteases profoundly inhibited cap-dependent translation [40]. Specific inhibition of HIV Pro reduced the extent of both necrosis and apoptosis in C8166 cells [41]. It was recently proposed that cleavage of RIPK1 by HIV-1 Pro might be one of several mechanisms by which HIV-1 counteracts host innate immune responses [33].

There is thus good evidence for cellular effects following expression of retroviral Protease. Although retroviral Protease is encoded in the human genome by HERV-K(HML-2) and expressed in the disease context, there is surprisingly little information as to potential functional relevance of HML-2 Pro expression. We therefore set out to identify human proteins processed by HML-2 Pro by employing specialized proteomics methods. Numerous human proteins were identified as substrates of HML-2 Pro. We further verified processing by HML-2 Pro for selected proteins in vitro and in vivo. Human proteins identified often exert various, often important cellular functions, and many of them are disease-relevant. The relevance of our findings for human disease is currently unknown, yet the sheer number of potentially disease-relevant proteins identified in our study as potential substrates of HML-2 Pro strongly argues for further specific analyses.

## Results

### Optimization of HERV-K(HML-2) protease activity

**Fig. 1:**
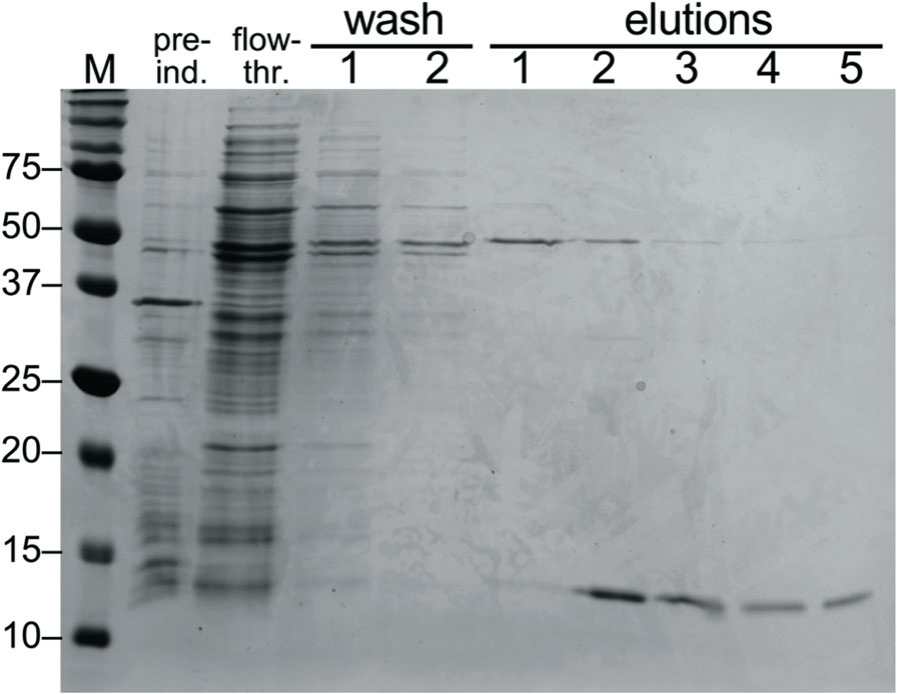
Purification of HERV-K(HML-2) Protease. A previously established method for purification of prokaryotically expressed HML-2 Pro was employed with minor modifications (see text). Samples were taken at various steps of the procedure, such as bacterial culture before induction (“pre-ind.”), flow-through (“flow-thr.”) after binding of bacterial lysate to Pepstatin A-agarose, two wash fractions, and 4 elution fractions. Proteins were separated by SDS-PAGE in a 15% PAA-gel and visualized by staining with Coomassie Blue. Molecular mass of marker proteins (M) are indicated on the left. Purified, auto-processed HML-2 Pro migrates at approximately 12 kDa

We sought to identify human cellular proteins that are substrates of HERV-K(HML-2) Pro, employing a modified Terminal Amine Isotopic Labeling of Substrates (TAILS) protocol [42, 43]. We first optimized HERV-K(HML-2) Protease activity prior to TAILS. We employed a cloned HML-2 Pro previously identified and shown to be enzymatically active [24]. Of note, the cloned Pro included self-processing sites and in-frame flanking sequence. HML-2 Pro was prokaryotically expressed and subsequently purified using a previously published protocol employing Pepstatin A, a specific inhibitor of retroviral aspartate proteinases, coupled to agarose beads [44]. In accordance with previous results, HML-2 Pro could be purified very efficiently and at relatively high yields (Fig. 1). As also observed before [44], HML-2 Pro self-processed from the precursor during the expression, purification, and renaturation steps (Additional file 2: Figure S1). We note that two different, enzymatically inactive mutants of HML-2 Pro (harboring mutations in catalytic motifs, see the Methods section) could not be purified due to inefficient binding to Pepstatin A-agarose (Additional file 2: Figure S1).

**Fig. 2:**
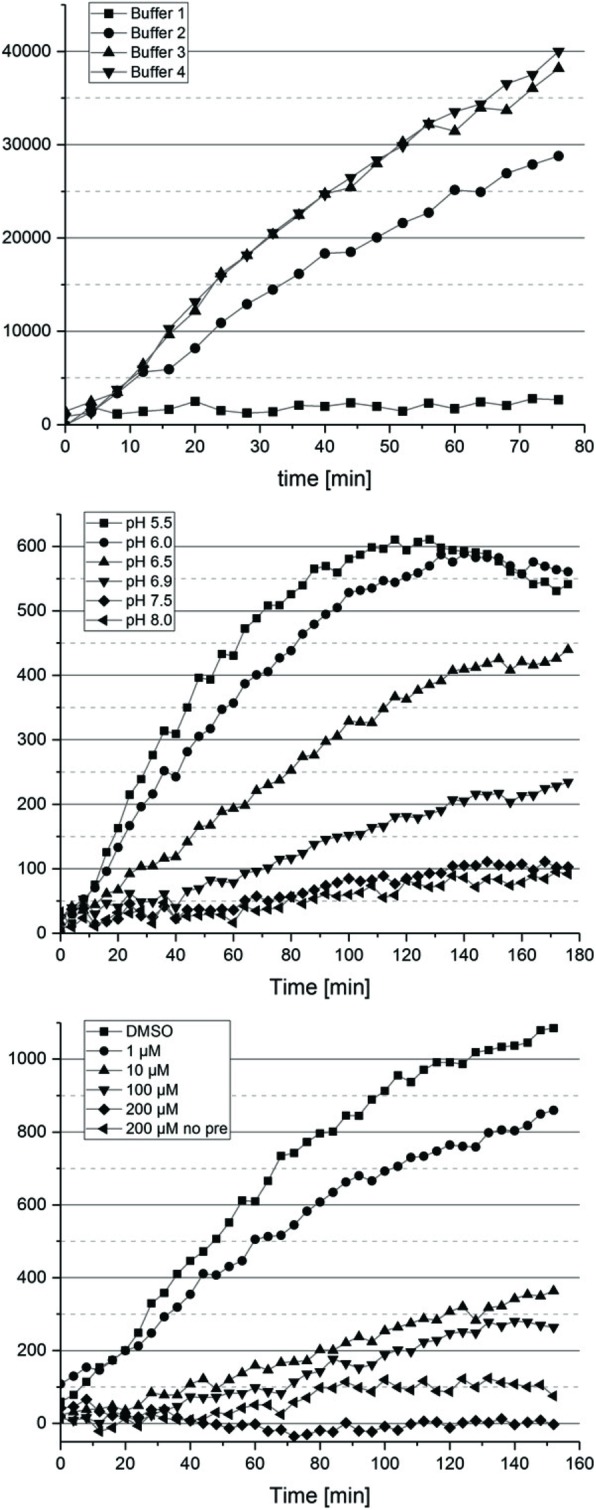
Optimization of reaction conditions of HERV-K(HML-2) Protease. Purified HML-2 Pro was incubated with a fluorescent anthranilyl-substrate and fluorescence emission was measured for the indicated time periods. Influence of different buffer compositions (top), pH values (middle), and Pepstatin A concentrations (bottom) on HML-2 Pro activity are depicted. Buffer compositions were as follows: Buffer 1: 20 mM PIPES, 100 mM NaCl, 1 mM DTT, 10% [v/v] Glycerol, pH 6.5; Buffer 2: 50 mM MES, 1 M NaCl, 20% [v/v] Glycerol, 1 mM EDTA, pH 5.0; Buffer 3: 50 mM MES, 1 M NaCl, 1 mM EDTA, pH 5.0; Buffer 4: 100 mM MES-TRIS, 1.25 M NaCl, pH 6.0. Effects of pH were measured in a buffer consisting of 100 mM MES, 1 M NaCl. Note the differing glycerol concentrations of buffers 2 and 3 (see the text). Also note that reactions at pH 5.5 and pH 6 depleted the substrate after approximately 110 min due to high HML-2 Pro activity. Effects of Pepstatin A at 200 μM were measured with and without pre-incubation of protease with Pepstatin A

Previous studies of HML-2 and other retroviral Proteases employed differing buffer systems and pH conditions when measuring HML-2 Pro activity (for instance, see [44, 45]). We therefore determined HML-2 Pro activity in various buffer systems using a fluorescent substrate previously shown to be processed by HIV Pro [46] and expected also to be processed by HML-2 Pro because of its very similar specificity profiles [47]. We found that HML-2 Pro displayed higher activity at conditions of high ionic strength. Higher concentrations of glycerol appeared to reduce HML-2 Pro activity (see the legend to Fig. 2), as did DMSO of 2% [v/v] and higher (not shown). Of further note, very similar HML-2 Pro activity was observed at different pH conditions for MES and PIPES-based buffer systems (not shown). A buffer composed of 100 mM MES and 1 M NaCl was chosen for lysis of HeLa cells and a PIPES-based buffer system was used for TAILS (see below). Further variation of reaction conditions between pH 5.5 and 8 established that HML-2 Pro was most active at pH 5.5 and somewhat less active at pH 6. Further reduced activity was seen for pH > 6, yet HML-2 Pro still displayed low activity at pH 8 (Fig. 2). In principle, these results are generally in accord with previous findings (for instance, see [20]). As further addressed below, several cellular compartments have an acidic pH of 6 or less [48].

Subsequent TAILS experiments involved Pepstatin A as an inhibitor of HML-2 Pro activity. We therefore also established the molar ratio required to effectively inhibit HML-2 Pro. We found that 200 μM Pepstatin A efficiently inhibited HML-2 Pro present at 460 nM. Inhibition was even more pronounced when reactions were pre-incubated with Pepstatin A for 10 min before addition of fluorescent substrate (Fig. 2).

### Identification of numerous human cellular proteins cleaved by HERV-K(HML-2) protease using TAILS

Previous studies indicated that retroviral aspartate Proteases, including HIV Pro, can process not only retrovirus-encoded proteins but also cellular proteins (see the Background section). We therefore were interested whether HML-2 Protease is also able to process human cellular proteins other than HML-2-encoded Gag protein. To do so, we employed a modified Terminal Amine Isotopic Labeling of Substrates (TAILS) procedure that identifies Protease-cleaved protein fragments by means of specific labeling and subsequent isolation of processed amine termini followed by mass-spectrometry [42, 43]. We incubated HeLa cell total protein lysate with purified HML-2 Pro employing established reaction conditions with regard to salt concentration, pH, and molar ratio of Protease and Pepstatin A (see above). HML-2 Pro-generated cleavage sites were subsequently identified by N-terminomics [43] using the TAILS approach. As a negative selection technique, TAILS is suitable for the analysis of natively blocked (e.g. acetylated) and natively free N-termini. Since proteolysis generates free N-termini, we focused on these species. During the TAILS procedure, free N-termini are chemically dimethylated.

**Fig. 3:**
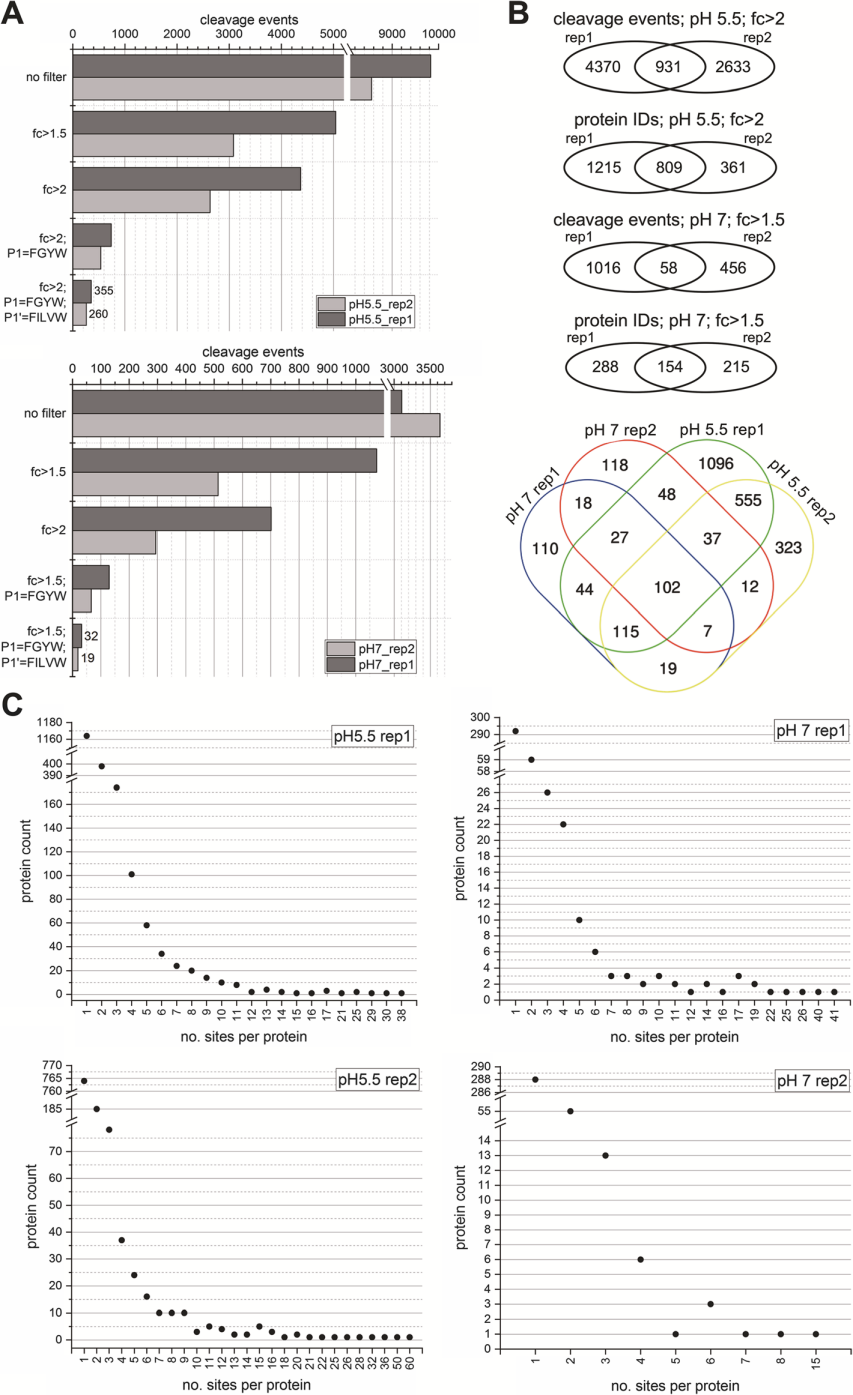
Cleavage sites in human proteins identified as substrates of HERV-K(HML-2) Protease by TAILS. **a**. Results of filtering of cleavage sites observed by TAILS. Results for two experiments (rep1, rep2) performed at pH 5.5 and pH 7 are each depicted. Various filters were applied, such as greater than 1.5-fold or 2-fold (fc) enrichment for the observed cleavage event compared to controls and particular amino acids in P1 and P1’ (see the paper text). Resulting numbers after applying the various filters are indicated by bars and by specific numbers when including P1 and P1’. **b**. Venn diagrams depicting overlap of cleavage sites and protein IDs in replicates (rep1, rep2) performed at pH 5.5 and pH 7. The overlap of protein IDs detected in all four experiments is depicted in the Venn diagram at the bottom. **c**. Numbers of cleavage sites in proteins identified as substrates of HERV-K(HML-2) Pro. Results are summarized for the replicate (rep1, rep2) TAILS experiments at pH 5.5 and pH 7. A single cleavage event was observed for the vast majority of proteins, fewer proteins were cleaved at more than one position, and a relatively small number of proteins were cleaved at up to 60 different positions within the particular protein. See Additional file 1: Table S2, for selected human proteins with multiple cleavage sites

We performed TAILS experiments at pH 5.5 and pH 7. As for the experiment at pH 5.5, TAILS identified greater than 8500 native free or proteolytically generated N-termini in both replicates 1 and 2 (Fig. 3, Additional file 1: Tables S1a,b). As an initial filter to discern background proteolysis from HML-2 Pro-dependent cleavage events, we selected those cleavage events that were enriched at least 2-fold upon HML-2 Pro incubation. We observed 4370 cleavage events in replicate 1 and 2633 cleavage events in replicate 2. A variation in protease activity, as well as the different methodological processing steps, may contribute to this variance. Of those, 931 cleavage events were common to both replicates (Fig. 3, Additional file 1: Tables S1a,b) and those corresponded to 548 different human proteins. For proteins cleaved in both replicates with at least 2-fold enrichment, yet not necessarily cleaved in the same position within a protein, we identified 2024 and 1170 unique protein IDs in the two replicates, respectively. Combining both replicates, 809 different human proteins showed replicated evidence of cleavage by HML-2 Pro (Fig. 3b, Additional file 1: Tables S1a,b). As implied by the above numbers, several human proteins showed multiple cleavage events per protein (Fig. 3c). For instance, we observed for Heat Shock Protein 90 Alpha Family Class B Member 1 (HSP90AB1) 30 and 50 cleavage events with at least 2-fold enrichment in different positions of the protein in replicates 1 and 2, respectively. For Myosin Heavy Chain 9 (MYH9), 25 and 60 cleavage events were observed, for Actin Beta (ACTB) 38 and 32, and for Heat Shock Protein Family A (Hsp70) Member 8 (HSPA8) 11 and 36, respectively (Additional file 1: Table S2).

Since protein degradation by HML-2 Pro may also occur in the cytoplasm or nucleoplasm at neutral pH rather than in acidic organelles, we also performed a TAILS experiment at pH 7. Overall, we observed fewer cleavage events, possibly due to lower enzymatic activity of HML-2 Pro at pH 7 (Fig.3a, Additional file 1: Tables S1c,d). Nevertheless, greater than 3100 native free or proteolytically cleaved N-termini were identified for replicates 1 and 2, respectively, of which 1074 (replicate 1) and 514 (replicate 2) cleavage events were enriched greater than 1.5-fold upon HML-2 Pro incubation, with an overlap of 58 cleavage events. For the pH 7.0 assay, we chose a less stringent cutoff value of 1.5-fold-change due to the lower activity of HML-2 Pro at pH 7.0. For a lower protease activity the TAILS approach may miss more potential substrates at the more stringent cutoff value of 2. Though, at the lower cutoff value the potential candidates have to be considered more carefully and additional experiments like the in vitro experiments and the experiments with cultured cells are of greater value. At a cutoff value of 1.5-fold-change, 442 (replicate 1) and 369 (replicate 2) different human proteins were affected by HML-2 Pro incubation. Combining the latter experiments, a total of 154 different human proteins showed replicated evidence of cleavage by HML-2 Pro at pH 7 (Fig. 3b). Of note, four human proteins were identified only in the pH 7 TAILS experiment, though with relatively low to medium enrichment of processing products (TAGLN: 3.8-fold; MAP1B: 4.1-fold; KTN1: 1.7-fold; EPB41L2: 1.6-fold).

Similar to the TAILS experiment at pH 5.5, we observed at pH 7 multiple cleavage events within several of human proteins enriched greater than 1.5-fold. For instance, there were 25 and 15 cleavage events in replicates 1 and 2, respectively, for HSP90AB1, 41 and 6 events for MYH6, 17 and 3 for ACTB, and 40 and 7 events for HSPA8 (Fig. 3c, Additional file 1: Table S2).

Combining all results, we identified 102 different human proteins cleaved by HML-2 Pro that were detected in all four TAILS experiments when applying 2-fold enrichment at pH 5.5 and 1.5-fold enrichment at pH 7 (Fig. 3b). We consider these findings to bear evidence of possible processing of human proteins by HML-2 Pro.

### Involvement of human proteins cleaved by HML-2 protease in diverse cellular processes

**Fig. 4:**
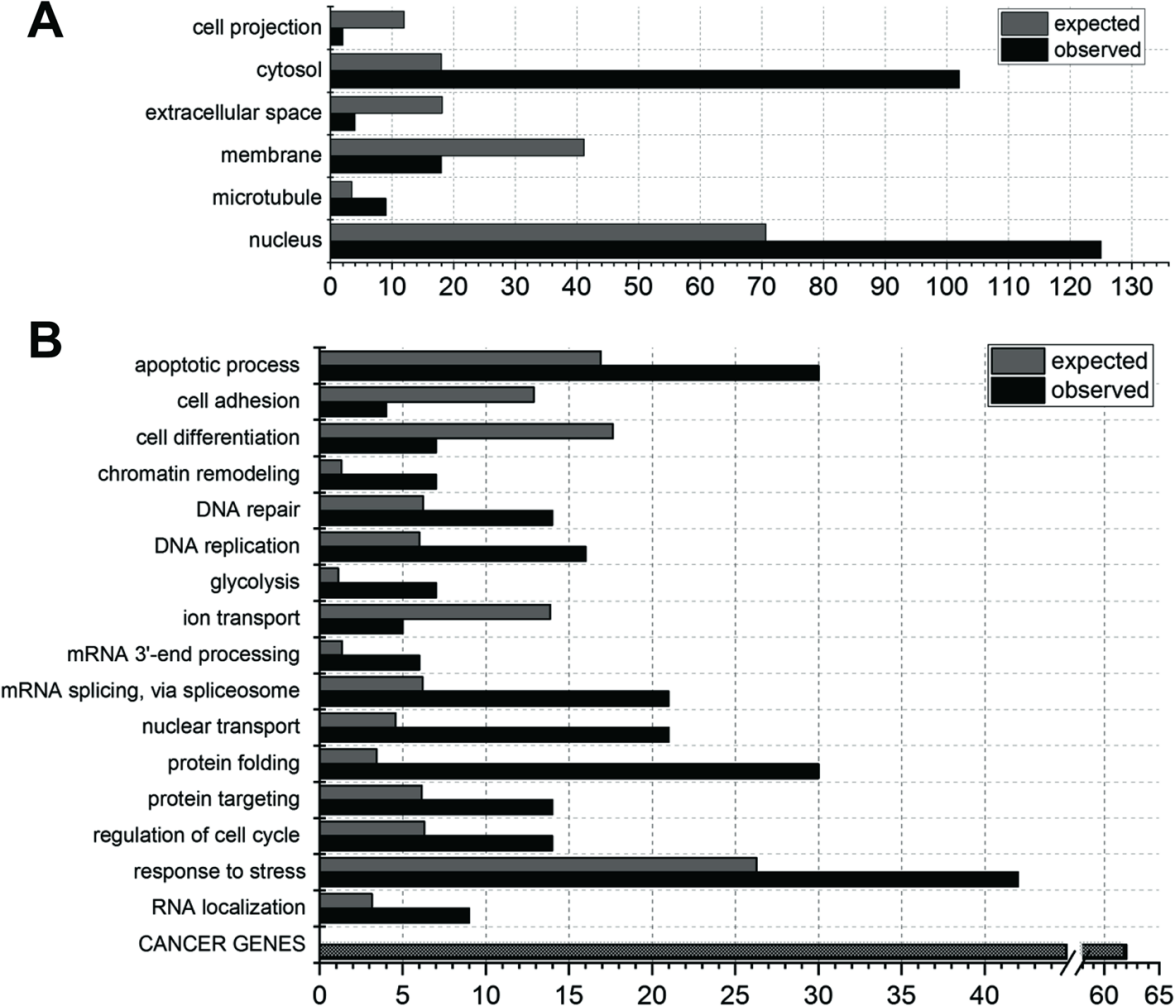
Gene Ontology term-based characteristics of human proteins identified as substrates of HERV-K(HML-2) Protease by TAILS. Selected cellular components (**a**) and biological processes (**b**) are depicted. Numbers were compiled using PANTHER (Protein ANalysis THrough Evolutionary Relationships) GO-Slim as provided at http://geneontology.org [51, 52]. Numbers of proteins per category expected by chance are also given. Graph (**b**) also depicts in the bottom-most bar the overlap of proteins identified by TAILS with cancer-relevant genes as compiled by COSMIC (Catalogue Of Somatic Mutations In Cancer; https://cancer.sanger.ac.uk/cosmic) [53]. See Additional file 1: Table S3 for COSMIC cancer genes

We next used the Gene Ontology (GO) database [49, 50] to identify biological properties of proteins identified by TAILS. Analysis of the 809 different human proteins common to the two pH 5.5 experiments indicated localization of proteins in diverse cellular compartments including cytosol, nucleus and membrane (Fig. 4a). Further GO term-based analysis of biological processes associated with the 809 human proteins showed their involvement in numerous biological processes, e.g. apoptosis, cell cycle regulation, DNA repair and replication, ion and nuclear transport (Fig. 4b). Moreover, intersection of the human genes corresponding to those 809 human proteins with genes included in the Catalogue Of Somatic Mutations In Cancer (COSMIC) database [53] identified 62 human genes/proteins in our dataset with an established relevance in oncology (Fig. 4b, Additional file 1: Table S3). Querying the Online Mendelian Inheritance in Man (OMIM) database [54] revealed genes for our dataset of 809 proteins to be associated with 265 different genetic disorder phenotypes, of which approximately 239 were described as inherited (Additional file 1: Table S4).

### Verification of cleavage of human proteins by HERV-K(HML-2) protease in vitro

We next sought to verify in vitro cleavage by HML-2 Pro of proteins identified by TAILS experiments. We focused on substrate candidates that were enriched more than 2-fold upon active HML-2 incubation in both replicates of the TAILS experiment at pH 5.5. A recent study profiled amino acid specificities of HERV-K(HML-2) Pro at aa positions P6–P1 and P1’–P6’ in, respectively, N-terminal and C-terminal direction with respect to the cleaved bond revealing, for instance, P1 as the major determinant of specificity and a preference for aromatic aa residues in P1 [47]. A subsequent profiling at pH 7 likewise revealed preferences for aromatic aa residues in P1 and aromatic and aliphatic aa residues in P1’ (data not shown). We utilized these published findings to reduce the list of candidate proteins by filtering for peptides from cleavage events having F, G, Y or W in P1, and F, I, L, V or W in P1’ (Fig. 3a). Furthermore, we selected proteins with a size compatible with an in vitro coupled transcription/translation system, and cellular localizations and biological functions based on associated GO terms. Eventually, we further analyzed 14 different human proteins (Table 1).

**Table 1:** Selected candidate proteins for verification of processing by HERV-K(HML-2) Protease^a^

Gene/Protein	Protein ID	Function	*ver.*
C15orf57 (CCDC32; Coiled-Coil Domain Containing 32)	Q9BV29	unknown	+/+
CALR (Calreticulin)	P27797	Prevents binding of glucocorticoid receptor to glucocorticoid response element. Important modulator of gene transcription. AD: Myelofibrosis With Myeloid Metaplasia; Somatic and Essential Thrombocythemia	−/n.t.
CIAPIN1 (Cytokine Induced Apoptosis Inhibitor 1)	Q6FI81	Cytokine-induced inhibitor of apoptosis. Anti-apoptotic effects in the cell. Negative control of cell death upon cytokine withdrawal	+/+
DDX3X (DEAD-Box Helicase 3, X-Linked)	O00571	Transcriptional regulation, mRNP assembly, pre-mRNA splicing, mRNA export, translation, cellular signaling, viral replication. Misregulation implicated in tumorigenesis. Cofactor for XPO1-mediated nuclear export of incompletely spliced HIV-1 Rev. RNAs. AD: Mental Retardation, X-Linked 102; Toriello-Carey Syndrome (Corpus Callosum, Agenesis Of, With Facial Anomalies And Robin Sequence)	i/n.t.
ENO1 (Enolase 1)	P06733	Role in glycolysis, growth control, hypoxia tolerance, allergic responses. Shorter isoform binds to c-myc promoter and functions as a tumor suppressor. Autoantigen in Hashimoto encephalopathy. AD: Cancer-Associated Retinopathy; Non-Herpetic Acute Limbic Encephalitis	i/n.t.
HSP90AA1 (Heat Shock Protein 90 Alpha Family Class A Member 1)	P07900	Proper folding of target proteins, regulation of transcription machinery. AD: Epidermolysis Bullosa Acquisita; Hypersensitivity Vasculitis	+/+
HSP90AB1 (Heat Shock Protein 90 Alpha Family Class B Member 1)	P08238	Signal transduction, protein folding and degradation; morphological evolution. AD: Bronchitis; Larynx Cancer	+/n.t.
MAP2K2 (Mitogen-Activated Protein Kinase Kinase 2)	P36507	Critical role in mitogen growth factor signal transduction. AD: Cardiofaciocutaneous Syndrome 4	+/+
PDIA3 (Protein Disulfide Isomerase Family A Member 3)	P30101	Modulates folding of newly synthesized glycoproteins. Molecular chaperone preventing protein aggregates. AD: Maxillary Sinus Squamous Cell Carcinoma; Anomalous Left Coronary Artery From The Pulmonary Artery	+/n.t.
PSMC4 (Proteasome 26S Subunit, ATPase 4)	P43686	26S proteasome assembly. AD: Cystic Fibrosis; Myotonia; Parkinson’s disease(?)	−/n.t.
RANBP1 (RAN Binding Protein 1)	P43487	Participates in regulation of cell cycle by controlling transport of proteins and nucleic acids into nucleus. May control progression through the cell cycle by regulating transport of protein and nucleic acids across nuclear membrane.	+/n.t.
RNASEH2A (Ribonuclease H2 Subunit A)	O75792	Component of RNase H2 complex that degrades RNA of RNA:DNA hybrids. Catalytic subunit of RNase H2. Mediates excision of single ribonucleotides from DNA:RNA duplexes. AD: Aicardi-Goutières Syndrome	n.t./+
RNASEH2B (Ribonuclease H2 Subunit B)	Q5TBB1	Component of RNase H2 complex. Non catalytic subunit of RNase H2. AD: Aicardi-Goutières Syndrome.	+/n.t.
S100A4 (S100 Calcium Binding Protein A4)	P26447	Regulation of cell cycle progression, differentiation. Altered expression implicated in tumor metastasis. AD: Bile Duct Adenocarcinoma; Pancreatic Cancer	−/n.t.
STUB1 (STIP1 Homology And U-Box Containing Protein 1)	Q9UNE7	Ubiquitin ligase/cochaperone. Targets misfolded chaperone substrates towards proteasomal degradation. Binds and ubiquitinates Hspa8 and Polb. Negatively regulates suppressive function of regulatory T-cells during inflammation. Negatively regulates TGF-β signaling. AD: Spinocerebellar ataxia, autosomal recessive 16; Gordon Holmes Syndrome	n.t./+
TUBA1A (Tubulin Alpha 1a)	Q71U36	Major component of microtubules. AD: Lissencephaly 3; Lissencephaly With Cerebellar Hypoplasia	+/+
TRIM28 (Tripartite Motif Containing 28)	Q13263	Nuclear corepressor for KRAB domain-containing zinc finger proteins. Mediates gene silencing. Important regulator of CDKN1A/p21. Mediates nuclear localization of KOX1, ZNF268, ZNF300. Required to maintain transcriptionally repressive state of genes in undifferentiated embryonic stem cells.	n.t./+

^a^Gene/Protein as given by approved gene/protein symbols and full names. A representative protein ID is given. Selected functional characteristics as well as associated diseases (“AD”) for respective genes/proteins were compiled from information provided by GeneCards [55]. Experimental verifications (“ver.”) of processing of particular proteins by HML-2 Pro in vitro and in vivo is indicated as “+/+”, no processing observed as “–”, inconclusive evidence as “i” (see the text), n.t.: not tested in either in vitro or in vitro experiments

We produced candidate proteins in vitro in a coupled transcription/translation system using either a radioactive label (^35^S-methionine) or a C-terminal HA-tag. We then incubated equal amounts of each candidate protein with purified HML-2 Pro, including a control reaction without Pro and one with Pro enzymatic activity inhibited by presence of Pepstatin A. Reactions were then subjected to SDS-PAGE followed by phosphorimager or Western blot analysis depending on the protein label.

**Fig. 5:**
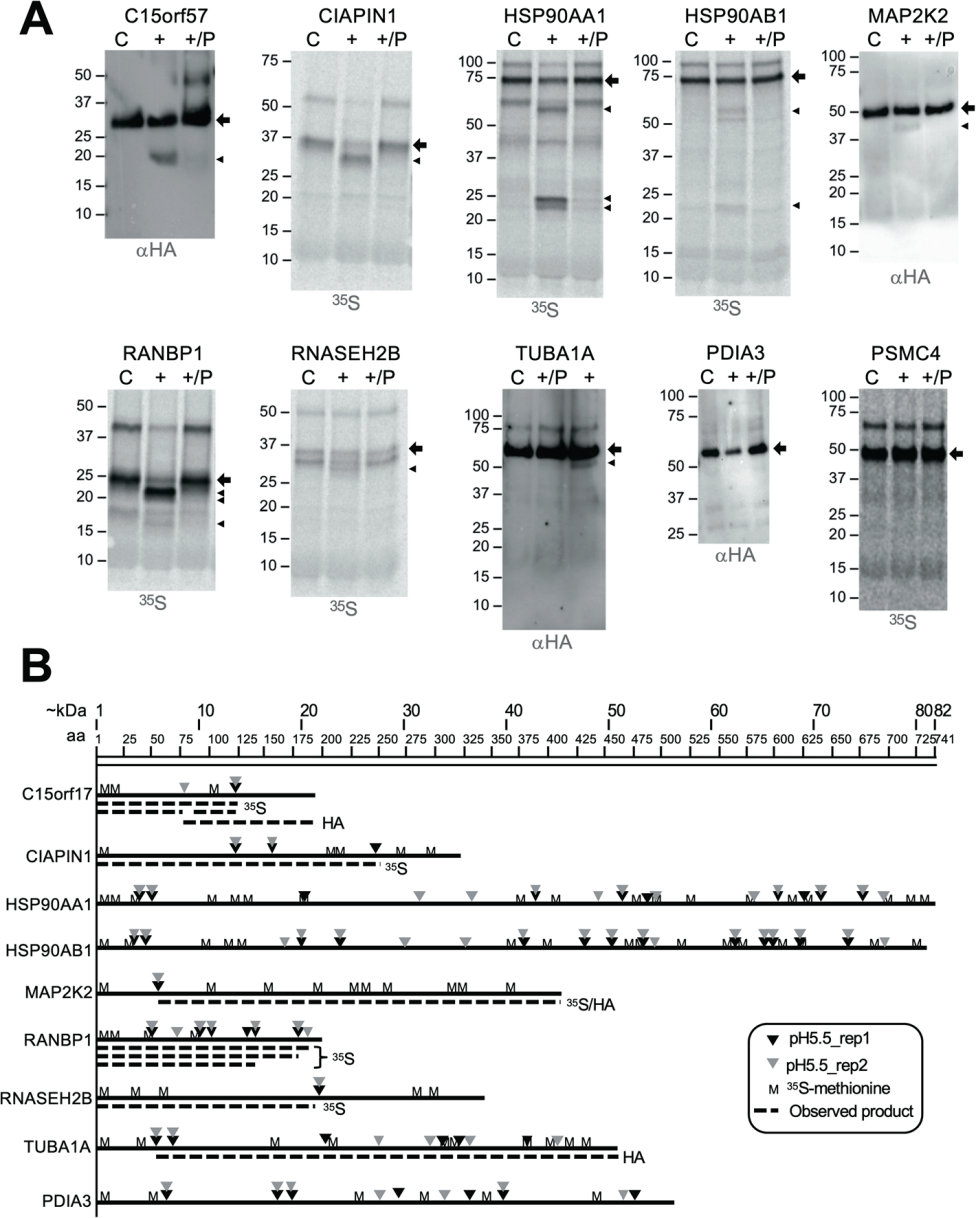
Verification of processing of human proteins by HERV-K(HML-2) Protease in vitro. Human candidate proteins were expressed in vitro using a coupled transcription/translation system. **a**. Results from protease incubations of various candidate proteins labeled with either ^35^S-methionine or a C-terminal HA-tag (“^35^S” and “HA”) are shown. Experiments included for each candidate protein a reaction without protease (“C”), one with protease (“+”), and one with protease and Pepstatin A (“+/P”). Reaction products were separated by SDS-PAGE in 10% PAA-gels and processed for phosphorimager analysis or HA-tag-specific Western blots depending on the label. Processing of full-length candidate proteins (indicated by an arrow) was evidenced by additional protein bands smaller than the respective full-length candidate protein (arrowheads) and/or a decrease in the amount of full-length candidate protein (see the Results section). One example of a candidate protein (PSMC4) without evidence of processing by HML-2 Pro is shown. **b**. Graphical depiction of candidate proteins confirmed to be processed by HML-2 Pro. The number of amino acids and corresponding molecular mass in kDa is indicated by scales at the top and by the line length for each protein. Positions of methionines and cleavage sites (grey and black arrowheads, respectively), as identified by TAILS in either one of the two replicate experiments at pH 5.5 (see the text), are indicated for each protein. Dashed lines indicate molecular masses of processing products observed experimentally for either ^35^S-methionine (“^35^S”)- or HA-tag (“HA”)-labeled candidate proteins. Note that the latter label will only detect C-terminal processing products. Processing products were not indicated for the two HSP90A proteins because observed products were difficult to assign due to too many observed cleavage sites. Processing of PDIA3 protein was supported by reduction of the amount of full-length protein, though no smaller processing products could be observed. Note that C15orf57 migrated slower in gel electrophoresis than predicted by molecular mass. See Additional file 2: Figure S2 for additional evidence of processing of candidate proteins by HML-2 Pro

Out of 14 different human proteins examined, we obtained evidence for processing by HML-2 Pro in vitro for 9 of those proteins. Evidence for processing included (i) a more or less reduced amount of full-length candidate protein compared to amounts of full-length protein in control reactions without Pro and with Pro plus Pepstatin A, (ii) presence of one or several additional protein bands in the reaction with Pro compared to the reaction without Pro, (iii) such additional protein bands also being present in the reaction with Pro plus Pepstatin A, yet at (much) lower amounts compared to the reaction with Pro. Different combinations of those criteria were observed in our verification experiments. In contrast, no or inconclusive evidence for processing by HML-2 Pro was obtained for 5 human proteins tested (Fig. 5a and Additional file 2: Figure S2).

TAILS experiments also provided information for actual cleavage site positions in candidate proteins. We found for 6 of the 14 different human proteins tested that HML-2 Pro had produced additional protein bands coinciding with sizes of processing products predicted by cleavage sites identified by TAILS (Fig. 5b).

### Verification of cleavage of human proteins by HERV-K(HML-2) protease in vivo

**Fig. 6:**
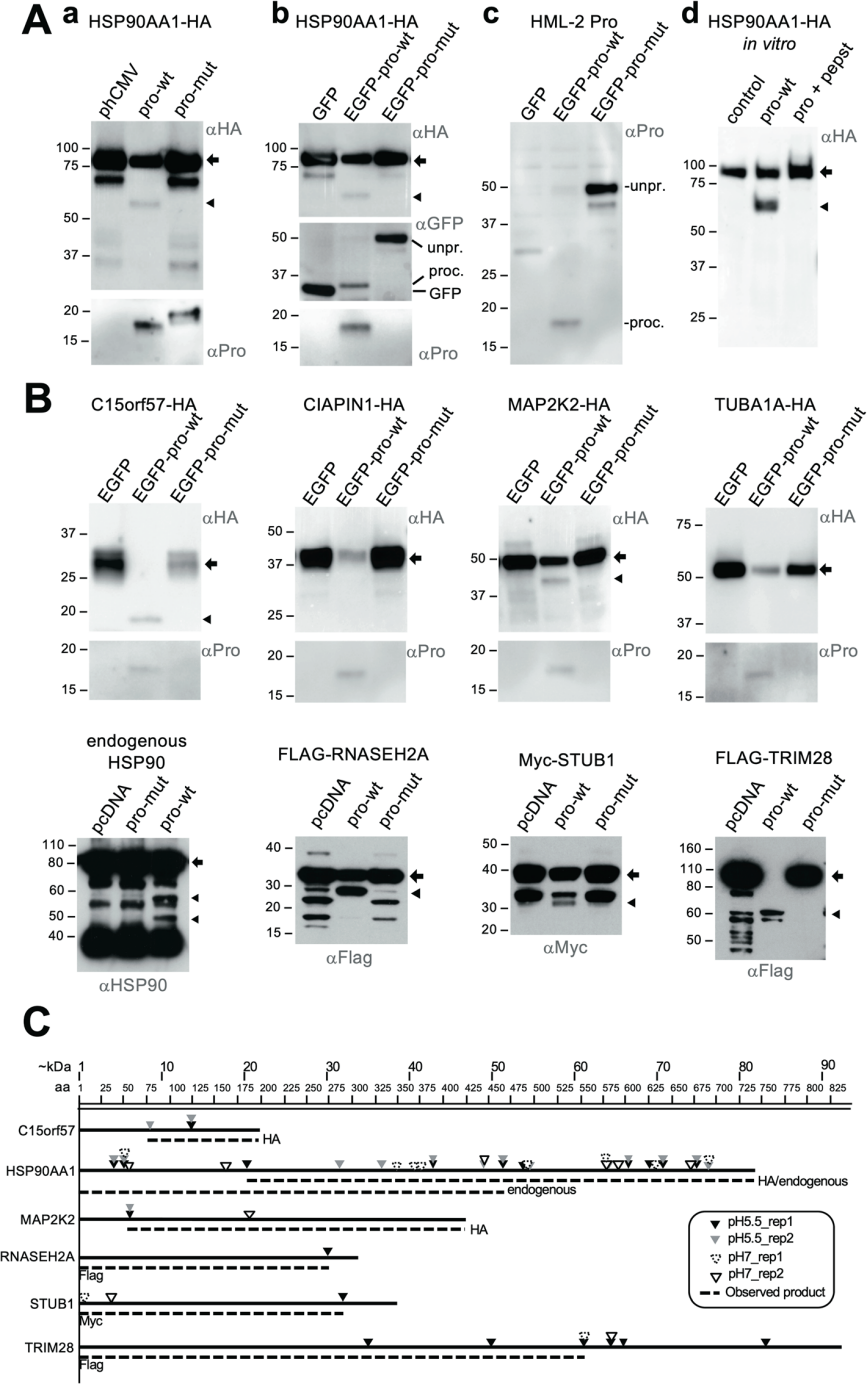
Verification of processing of human proteins by HERV-K(HML-2) Protease in vivo. Human candidate proteins and HML-2 Pro were co-expressed in HeLa cells in vivo and detected by Western blot using antibodies as indicated. For each blot, the leftmost lane is a control co-transfected with a plasmid encoding a candidate protein and either a GFP-encoding plasmid or empty phCMV, pcDNA6 myc/his B, or pcDNA5 FRT/TO vector, depending on GFP-Pro or (sole) Pro co-expressed in the experiment (see below). Candidate protein co-expressed with wild-type Pro (pro-wt) and mutant Pro (pro-mut) were loaded in lanes 2 and 3 each. Pro was expessed as either (sole) Pro or EGFP-Pro. Blots were probed with α-HA, α-GFP, α-Pro, or an α-HSP90 antibody as indicated. Full-length candidate protein and processing products are indicated by arrows and arrowheads, respectively (see below). **A** Representative results from control experiments co-expressing HSP90AA1 with either HML-2 Pro or EGFP-Pro. Relevant blot regions are shown. When expressing pro-wt and pro-mut, HML-2 Pro can be detected as approximately 18 kDa and 19 kDa protein bands representing self-processed and unprocessed products, respectively, Pro (**a**, bottom blot). When HML-2 Pro is expressed as EGFP-Pro-wt or EGFP-Pro-mut fusion protein, proteins of approximately 30 kDa and 47 kDa, representing processed and unprocessed EGFP(−Pro) can be detected with an α-GFP antibody (**b**, middle blot). Unprocessed EGFP-Pro(−mut) and self-processed Pro of approximately 50 kDa and 18 kDa, respectively, can be detected when using an α-Pro antibody (**b**, bottom blot; **c**). **B.** Selected Western blot results from co-expression of candidate proteins and HML-2 Pro. Candidate proteins were tagged with N- or C-terminal epitopes and detected with respective epitope-specific antibodies as indicated. Note the more or less complete reduction of amounts of full-length candidate protein (arrows), and sometimes processing products (arrow heads), in lanes with co-expressed HML-2 Pro. Note in panel **Aa** and **Ab** that the same processing product was detected for HSP90AA1 in vitro and in vivo (the HSP90AA1 in vitro result is shown again in **Ad** for the sake of convenience). Also compare in vitro and in vivo results for C15orf57 and MAP2K2 as additional examples of similar sized processing products. Molecular masses of co-migrating marker proteins are indicated. Note that the α-Pro Western blot result shown for CIAPIN1-HA is extracted from the Western blot shown in **Ac**. See Additional file 2: Figure S3 for loading controls as well as more examples of proteins processed by HML-2 Pro in vivo. **C.** Graphical depictions of candidate proteins and predictions of processing products as observed when co-expressing candidate proteins and HML-2 Pro-wt in vivo. Numbers of amino acids and corresponding molecular mass (kDa) are indicated by scales at the top and by the length of lines for each protein. Positions of cleavage sites, as identified by TAILS experiments at pH 5.5 and pH 7, are indicated by triangles for each protein. Dashed lines indicate molecular masses of processing products and take into account whether the candidate protein was expressed with an N-terminal or a C-terminal epitope tag. Note the overlap between predictions and molecular masses of processing products observed in vivo

We also investigated candidate proteins for their ability to be processed in vivo. We selected proteins confirmed in vitro as substrates of HML-2 Pro, along with proteins identified by TAILS that were of functional interest and readily available to us as cloned cDNAs. We co-expressed in HEK293T cells epitope-tagged candidate proteins together with wild-type (enzymatically active) or mutant (inactive) HML-2 Pro, with or without an enhanced green fluorescent protein (EGFP) tag, and performed Western blot analysis with epitope-tag-specific antibodies. Expression of Pro was detected using either a polyclonal α-HML-2 Pro antibody [19], or an α-EGFP antibody (kindly provided by Gabriel Schlenstedt, University of Saarland). The α-Pro pAb detected proteins of sizes expected for self-processed and (unprocessed) precursor forms of both wild-type and mutant HML-2 Pro. The α-EGFP antibody detected proteins of sizes expected for EGFP-Pro precursor and the EGFP portion after auto-processing of Pro (Fig. 6a). Importantly, processing of HML-2 Pro from an EGFP-Pro fusion protein provides further strong experimental support for HML-2 Pro becoming active independent of retroviral particle formation and budding.

For many of the tested candidate proteins, we observed pronounced reduction of the amount of full-length candidate protein, in some instances to below detection limits (Fig. 6a, b and Additional file 2: Figure S3). Notably, for some of the tested candidate proteins, additional products smaller in size than the full-length proteins were detected when co-expressing wild-type but not mutant HML-2 Pro, specifically for C15orf57-HA, HSP90-HA, MAP2K2-HA, FLAG-TRIM28, FLAG-RNASEH2A, and Myc-STUB1 (Fig. 6a, b). For three proteins, sizes of such additional products were very similar to sizes of cleaved protein products detected in the in vitro verification experiments. Specifically, a fragment of 15 kDa was seen for C15orf57-HA. A fragment of 60 kDa was also detected for HSP90 by both the anti-HA and the anti-HSP90 antibodies. The latter antibody also detected an approximately 50 kDa fragment of HSP90. A fragment of approximately 42 kDa was also detected for MAP2K2-HA (compare Figs. 5a, 6a, b). This suggests that HML-2 Pro processing of these candidate proteins in vivo reproduced the same (more or less stable) processing products as the in vitro reactions. Truncated protein products were also detected by Western blotting of additional candidate proteins tested only in vivo, specifically 62 kDa, 30 kDa, and 31 kDa bands for FLAG-TRIM28, FLAG-RNASEH2A, and Myc-STUB1, respectively (Fig. 6b). Thus HML-2 Pro-mediated processing of these proteins also appears to produce stable processing products.

Importantly, and similar to the in vitro verification experiments, the sizes of additional protein products observed coincided well with the sizes predicted by cleavage sites identified in TAILS experiments (Fig. 6c).

### Degradation of candidate proteins is not due to HML-2 protease induced cell death

When expressing HML-2 Pro in HEK293T and HeLa cells, we noted under the microscope cell death for a relatively small proportion of cells. The amount of cell death seemed higher for HeLa than for HEK293T cells. No such cell death was observed when expressing mutant HML-2 Pro. Cell death appeared reduced in the presence of 1 μM indinavir, a strong inhibitor of HIV Pro, and with less potency against HML-2 Pro in cell culture (not shown) [20, 56].

We therefore quantified by FACS analysis the relative amount of cell death following HML-2 EGFP-Pro expression in HEK293T cells. We determined relative numbers of EGFP-, thus Pro-expressing cells at 5, 10, 24, 30, and 48 h after transient transfection with plasmids encoding either EGFP, EGFP-Pro-wt, or EGFP-Pro-mut. Approximately 60% of gated live cells expressing EGFP-Pro-wt or EGFP-Pro-mut were EGFP-positive up to 48 h post-transfection, indicating that only a minority of cells expressing HML-2 Pro are driven into cell death over the course of our expression experiments (Additional file 2: Figure S4).

HIV Pro has also been reported to induce apoptosis (see the Background section). Various cellular proteins are degraded during apoptosis due to activation of caspases [57]. We therefore asked whether observed cleavage of candidate proteins by HML-2 Pro could also be attributed to cleavage by caspases. We transiently expressed proteins HSPA90AA1-HA, MAP2K2-HA and C15orf57-HA in HEK293T cells and subsequently induced apoptosis by addition of Staurosporin at 2 μM. HEK293T cells harvested after 5 h did not show evidence of processing of candidate proteins due to apoptotic processes (Additional file 2: Figure S4). Importantly, a processing product of a size observed when co-expressed with HML-2 Pro was not visible (Fig. 6a, b). Of further note, in the case of HSP90AA1-HA and MAP2K2-HA co-expressed with HML-2 Pro, addition of a pan-caspase inhibitor (Q-VD, 25 μM) did not reduce the amount of processing product observed, but rather increased it slightly when compared to control cells expressing HML-2 Pro in the absence of Q-VD (Additional file 2: Figure S4).

### Several HERV-K(HML-2) loci in the human genome potentially encode active protease

**Fig. 7:**
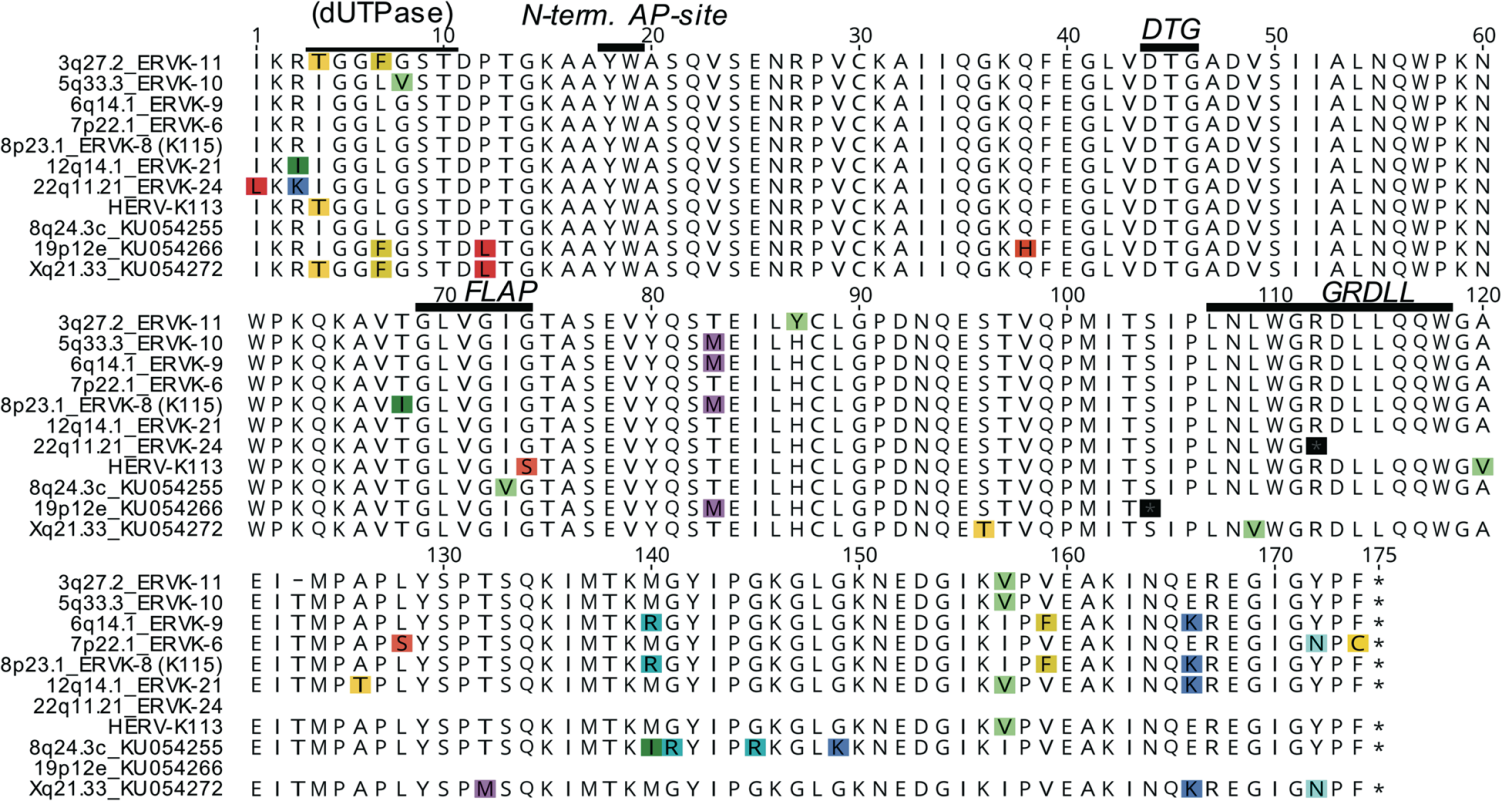
Multiple alignment of amino acid sequences of Proteases potentially encoded by HERV-K(HML-2) loci. Because HML-2 Pro is translated via a ribosomal frameshift from the Gag ORF only HML-2 Pro sequences that also harbor a full-length Gag ORF are included. Note that other HML-2 loci may also encode protease in the case of translation bypassing Gag-Pro frameshifts. The HML-2 Pro ORF also encodes an upstream dUTPase. The C-terminal “last” dUTPase motif is included in the multiple alignment. Also indicated are a previously reported N-terminal auto-processing site for HML-2 Pro [44], and DTG, FLAP and GRDLL motifs conserved in retroviral aspartyl proteases. Note the early stop codons in two sequences that partially or entirely remove the GRDLL region. The HML-2 locus designations used here are a combination of two established naming systems; the first based on the location of HML-2 loci in chromosomal bands [58] and the second based on HUGO Gene Nomenclature Committee (HGNC)-approved designations of transcribed HML-2 loci [59]. HERV-K113 and the three bottom-most sequences are HML-2 sequences not present in the human reference genome [2, 60]. Also note that locus chr3q27.2_ERVK-11 harbors a fused Gag-Pro ORF that extends approximately 700 aa in the N-terminal direction. Locus 7p22.1_ERVK-6 represents the protease sequence used for in vitro and in vivo experiments in this study

We were interested in which HML-2 loci in the human genome may produce an active Protease when they are transcribed and translated in a retroviral fashion, that is, the Pro ORF is translated via ribosomal frameshift between the Gag and Pro ORFs. Therefore, we examined HML-2 locus sequences in the human reference genome sequence, as well as among HML-2 sequences previously reported as missing from the reference genome, for presence of Gag and Pro ORFs. We subsequently predicted sequences of encoded Pro proteins for HML-2 loci fulfilling those criteria (Fig. 7). We identified 6 different HML-2 loci in the human reference genome (3q27.2_ERVK-11; 5q33.3_ERVK-10; 6q14.1_ERVK-9; 7p22.1_ERVK-6; 8p23.1_ERVK-8 (K115); 12q14.1_ERVK-21) potentially capable of translating a Pro protein of canonical length. None of the corresponding protein sequences displayed amino acid alterations within the conserved catalytic DTG, FLAP and GRDLL motifs (Fig. 7). Of note, HML-2 locus 3q27.2_ERVK-11 displayed a fused Gag-Pro ORF extending approximately 700 aa in the N-terminal direction. Another HML-2 locus (22q11.21_ERVK-24) displayed a premature stop codon in the conserved GRDLL motif. Three out of four non-reference HML-2 sequences displayed full-length ORFs, yet one of them harbored a G → S change and another an I → V change within the FLAP-motif (Fig. 7).

## Discussion

Retroviral aspartyl proteases are known to process various cellular proteins that are not directly correlated with or important for the retroviral replication cycle. Processing of such cellular proteins does not appear to play a major role during replication of exogenous retroviruses and may be regarded rather as cellular side effects of infections by exogenous retroviruses. However, processing of cellular proteins by retroviral proteases may be much more critical when the protease is encoded by endogenous retroviruses that are stable, vertically inherited components of a genome. In fact, the HERV-K(HML-2) group encodes active protease and HML-2 transcription and expression of HML-2 proteins has been reported to be upregulated in various human cancers, sometimes early in cancer development, such as in GCT carcinoma in situ [8]. Importantly, there is strong evidence that active HML-2 Pro is expressed in tumor cells and tumor-derived cell lines. HML-2 encoded retroviral particles budding from GCT cell lines were shown 25 years ago (for instance, see [22]). Large amounts of processed HML-2 Gag protein are present in GCT tissue and processed HML-2 Gag protein was furthermore demonstrated in GCT cell lines and tissue samples (for instance, see [10, 22, 23]). HML-2 Pro appears to become activated, and is thus present in cells, independent of budding of retroviral particles (see the Background section and below). The cellular consequences of expression of active HML-2 Pro are currently unknown. The disease relevance of HML-2 Pro is therefore unknown as well.

We employed a recently developed strategy, TAILS, for identification of human cellular proteins that are potential substrates of HML-2 Pro using purified HML-2 Pro and the proteome of HeLa cells as a model system. Our analysis identified a surprisingly high number—at least in the hundreds—of human proteins as potential substrates. A different positional proteomics approach recently identified more than 120 human proteins as processed by HIV-1 Pro in vitro [30]. Our experimental approach can be expected to be more sensitive and to thus identify more proteins than the approach employed in that study. Interestingly, 57 proteins identified in our study were also identified in that study [30] likely because of overall similar specificity profiles of HIV-1 and HML-2 Pro [47]. Methodologically our study comprised a broad TAILS approach followed by in vitro experiments and experiments in cultured cells of selected proteins. This study is a valuable example of this method combination for providing insight into potential substrates of yet under-investigated proteases.

The number of human proteins processed by HML-2 Pro in vivo is currently difficult to estimate with certainty. The two TAILS experiments at pH 5.5 identified approximately 4300 and 2600 cleavage events with at least 2-fold enrichment of a cleavage event of which 809 different human proteins were common to both experiments. Although HML-2 Pro displayed overall lower activity at pH 7, we still identified 500 to 1000 cleavage events with greater than 1.5-fold enrichment involving 154 different human proteins cleaved in both pH 7 experiments.

Furthermore, we verified processing by HML-2 Pro for 9 out of 14 (65%) human proteins in vitro. The great majority of the different human proteins examined in vivo also showed evidence of processing by HML-2 Pro. Our selection of candidate proteins for in vitro testing involved a filter for certain amino acids in positions P1 and P1’ of observed cleavage sites, and a specific molecular mass range due to technical limitations of the experimental in vitro transcription/translation system used for verification. Even when assuming favored amino acids in positions P1 and P1’ to be required for cleavage by HML-2 Pro, one still has to consider several hundred human proteins as potentially processed by HML-2 Pro (see Fig. 3a). Furthermore, our TAILS analyses examined human proteins expressed in HeLa cells. Preparation of protein lysates likely involved systematic loss of some protein species because of inadequate lysis conditions for those proteins, thus very likely resulting in an incomplete sampling of the HeLa proteome. Our analysis likely also missed human proteins expressed at very low levels, or not at all, in HeLa cells. A recent study of NCI-60 cell lines identified ~ 5600 human proteins as the core (cancer) proteome, another ~ 5000 proteins showing a more distinct expression pattern between tissues, and ~ 2000 proteins to be cell line- or tissue-specific and not part of the core proteome [61]. Therefore, TAILS experiments utilizing cell lines other than HeLa can be expected to identify a considerable number of additional proteins as (candidate) substrates of HML-2 Pro. We therefore hypothesize that even more human proteins than observed in our experiments are potential substrates of HML-2 Pro.

We verified processing by co-expressed HML-2 Pro for about two-thirds of selected candidate proteins in vitro and the great majority in vivo. For the latter, levels of processing ranged from slight to complete reduction of full-length candidate protein, sometimes accompanied by (more or less) stable presumed processing products. We conclude that observed reduction of full-length candidate proteins was not due to cell death (potentially apoptosis, see the Background section) triggered by HML-2 Pro and activation of caspases that then process candidate proteins. FACS analysis indicated that the majority of transfected cells were still alive at up to 48 h. Therefore, expressing HML-2 Pro does not inevitably cause cell death. Furthermore, apoptosis triggered by Staurosporin neither reduced amounts of full-length candidate protein nor generated smaller processing products, as is the case when expressing HML-2 Pro. It was furthermore reported previously that an HA-tag can be cleaved by caspase-3 and -7, causing loss of immunoreactivity for HA-tagged proteins [62]. We ruled out the possibility that observed loss of HA-tagged candidate proteins in our experiments is due to such HA-tag processing. First, our in vitro experiments demonstrated processing of candidate proteins by HML-2 Pro, the specificity of which was further demonstrated by reduced processing in the presence of Pepstatin A. Second, reduced levels of full-length protein were also observed for candidate proteins carrying epitope-tags other than HA. Third, FACS data show that the majority of HML-2 Pro expressing cells are still alive after > 30 h, thus apoptosis was not triggered in those cells. Fourth, induction of apoptosis by Staurosporin, together with activation of caspases (− 3 and − 7), did not reduce amounts of HA-tagged full-length candidate protein. Fifth, while cell death observed for HIV Pro was described as apoptosis [38, 63], the specific mechanism through which HML-2 Pro-expressing cells die remains to be investigated. Our findings indicate that caspase-3 is present at only low amounts in HML-2-Pro-expressing cells (not shown).

Our findings strongly argue for HML-2 Pro being enzymatically active in vivo and further corroborate processing of human proteins by HML-2 Pro in TAILS experiments at pH 7. There is additional evidence for HML-2 Pro being enzymatically active in vivo as indicated, for instance, by processing of HML-2 Gag protein in vivo (see above). HIV-1 Pro has been detected in membranes, mitochondria and cytoplasm, and was also shown to be active in the cytoplasm [34, 63]. Our analysis identified numbers of putative cleaved human proteins that localize to cytosol, membrane, mitochondria, and other organelles, based on GO-terms. We found EGFP-tagged mutant HML-2 Pro, that is unable to self-process from the EGFP-tag, to localize strongly to the nucleus, as well as in the cytoplasm of U2OS osteosarcoma cells and HEK293T cells when examined by fluorescence microscopy (Additional file 2: Figure S5). Likely, HML-2 Pro also localizes to the cytosol in cell types other than U2OS and HEK293T and thus could process proteins localizing to, or trafficking through, the cytosol. Whether HML-2 Pro also localizes to, and is active in, other cellular compartments remains to be investigated. HML-2 Pro likely would be enzymatically most active in compartments such as secretory granules, late endosomes and lysosomes for which pH 4.7 to 5.5 has been reported [48].

Human proteins identified as substrates of HML-2 Pro participate in a diverse array of cellular processes as assessed by GO-term analyses [49, 50]. Our GO-term analyses served to compile biological information on proteins identified in our proteomics experiments. Approximately 5 times more cytosolic proteins and 2 times more nuclear proteins than expected by chance were identified in TAILS experiments. However, it currently appears unlikely that HML-2 Pro preferentially processes respective proteins. Human proteins identified as substrates of HML-2 Pro furthermore considerably overlap with cancer-relevant genes based on COSMIC (Catalogue Of Somatic Mutations In Cancer) [53] and with Mendelian disease phenotypes as revealed by OMIM [54] (Additional file 1: Tables S3, S4). HML-2 Pro expression might thus impact cell biology in various ways and contribute to disease by affecting one or more cellular processes.

HML-2 Gag-Pro precursor protein, from which Pro self-processes, is translated via an occasional ribosomal frameshift between Gag and Pro ORFs. Compared to Gag, lesser amounts of Pro are thus likely produced in cells. For the purposes of our validation experiments, we expressed HML-2 Pro from a subregion of the Pro ORF from which Pro self-processed. The actual amounts of enzymatically active Pro in cells and tissues expressing HML-2 are currently unknown. However, since HML-2 Pro is an enzyme, a relatively small amount of active Pro could have a significant impact on cell biology when expressed over long periods of time. Preliminary data indicate that HML-2 Pro is detectable by a rabbit polyclonal anti-HML-2-Pro antibody [19] in cell lines known to overexpress HERV-K(HML-2) (Additional file 2: Figure S6). Furthermore, transient expression of EGFP-Pro-mut (Pro does not self-process from the precursor, see Fig. 6Ab) in such cell lines results in processing of the Pro portion. The amount of such processing can be reduced in the presence of HIV Pro inhibitor Indinavir (Additional file 2: Figure S6). It thus can be concluded that active HML-2 Pro that is present in those cells processes EGFP-Pro-mut.

Without further specific experiments, the cellular consequences deriving from processing of many human proteins by HML-2 Pro remain speculative. Our identification of proteins as potential substrates for HML-2 Pro processing lays groundwork for a number of specific experiments. Disease conditions involving known or suspected HERV-K(HML-2) misregulation or upregulation should be of greatest interest, including, for example, some cancers and amyotrophic lateral sclerosis [7, 64]. To the best of our knowledge, a functional role for HML-2 Pro in such diseases has not yet been explored.

Expression of HML-2 Pro in disease conditions will depend on which HERV-K(HML-2) loci are transcribed as only a subset of HML-2 loci appears capable of producing active protease. Our analysis indicated 6 currently known reference and 1 non-reference HML-2 sequences potentially capable of producing active protease. Alleles affecting Gag and Pro ORFs were previously shown for locus 7p22.1_ERVK-6 [65], thus only certain alleles of that locus would encode active protease. Hitherto unidentified alleles of some other HML-2 loci likewise may possess protease-coding capacity. It is also conceivable that some Pro ORFs are translated without Gag-Pro ORF ribosomal frameshifts or through translational starts within C-terminal Gag ORF portions. Frameshift-causing pseudoknot RNA structures may also influence protease-coding capacity of HML-2 loci. In any case, consideration of HML-2 Pro in a particular disease should include identification of HML-2 loci actually transcribed along with their protease coding capability. For instance, Pro-encoding HML-2 loci with a Gag ORF, specifically loci 3q27.2_ERVK-11, 5q33.3_ERVK-10, 6q14.1_ERVK-9, 7p22.1_ERVK-6, and 8p23.1_ERVK-8 (see Fig. 7), were previously identified as transcribed in GCT tissues and/or the GCT-derived cell line Tera-1 [66–68]. Loci 5q33.3_ERVK-10 and 7p22.1_ERVK-6 were identified in the context of amyotrophic lateral sclerosis, yet factual overexpression of HERV-K(HML-2) in ALS is currently debated [69–72].

We further note that our findings may also have implications for better understanding biological consequences of certain non-human endogenous retroviruses. For instance, endogenization of Koala endogenous retrovirus (KoRV) in Koalas (*Phascolarctos cinereus*) is ongoing, and KoRV-positive animals develop serious, life-threatening diseases, in particular malignant neoplasias [73]. The mechanism(s) of KoRV viral pathogenesis is poorly understood. One might hypothesize that disease-relevant Koala cellular proteins are processed by KoRV-encoded protease thus contributing to disease development.

Taken together, our findings for HERV-K(HML-2) Pro call for further experiments to better understand the relevance of endogenous retrovirus-encoded protease in health and disease in human and other species.

## Conclusions

Retroviral proteases are known to process cellular proteins. While functionally less relevant in the case of expression of exogenous retroviruses, constitutive expression of protease encoded by an endogenous retrovirus is potentially more consequential if processing of cellular proteins affects cell physiology. Employing specialized proteomics technologies followed by additional experimental verification, we suggest that retroviral protease of disease-associated human endogenous retrovirus HERV-K(HML-2) processes numerous cellular proteins in vitro and in vivo, with many of those proteins known to be disease-relevant. Deregulated transcription of HERV-K(HML-2), as reported for various human diseases, could result in expression of HERV-K(HML-2) Protease and consequent processing of various cellular proteins with unknown physiological consequences and disease relevance. Our study provides an extensive list of human proteins potentially deserving further specialized investigations, especially relating to diseases characterized by deregulated HERV-K(HML-2) transcription. Disease-relevance of endogenous retrovirus-encoded protease may also be considered in non-human species.

## Methods

### Plasmid constructs for prokaryotic and eukaryotic protease expression

We generated plasmid constructs for prokaryotic expression of HERV-K(HML-2) protease (Pro). The coding region, including flanking sequence regions and self-processing sites of enzymatically active HERV-K(HML-2) Pro, as encoded by the previously described HERV-K(HML-2.HOM) provirus (nt 3277–3769; GenBank acc. no. AF074086.2) [24], was cloned in-frame into pET11d prokaryotic expression vector (Novagen). To do so, the particular region was amplified by PCR from a HERV-K(HML-2.HOM) provirus previously cloned in pBluescript [24]. The forward PCR primer added an *Nhe*I site and the reverse primer added a stop codon and a *Bam*HI site to the PCR product. The PCR product was subcloned into pGEM T-Easy vector (Promega). The insert was released by an *Nhe*I/*Bam*HI digest and cloned in-frame into *Nhe*I/*Bam*HI-digested pET11d plasmid (Novagen) giving rise to pET11dPro.

For eukaryotic Pro expression, nt 3415–3946 of HERV-K(HML-2.HOM) were amplified by PCR, with the forward primer adding a *Bam*HI site, a spacer and a Kozak consensus sequence and the reverse primer adding a *Bam*HI site. The PCR product was likewise subcloned into pGEM T-Easy vector, followed by release of the insert by a *Bam*HI digest and cloning into a *Bam*HI-digested phCMV eukaryotic expression vector, giving rise to phCMV-Pro-wt.

For the eukaryotic expression of EGFP-pro fusion protein nt 3415–3946 of HERV-K(HML-2.HOM) were amplified by PCR, with both forward and reverse primers adding a *Bam*HI site each. The PCR product was subcloned into pGEM T-Easy vector, followed by release using *Bam*HI and cloning into *Bam*HI-digested pEGFP-C1 in frame with the EGFP ORF, giving rise to pEGFP-Pro-wt.

Note that the HERV-K(HML-2.HOM) Pro region used for generation of expression vectors included a known N-terminal auto-processing site [20], thus allowing for release of active HERV-K(HML-2.HOM) Pro from a precursor protein, e.g. EGFP-Pro.

Following the cloning strategies used for the design of wild-type Pro containing-plasmids, we also generated plasmids containing a mutated protease, specifically pET11dPro-mut, phCMV-Pro-mut, and pEGFP-Pro-mut. Enzymatically inactive Pro variants were generated by PCR using Phusion polymerase (New England Biolabs) and wt-Pro in pGEM T-Easy vector as the template, followed by re-ligation of PCR products. One of the two PCR-primers introduced the desired mutation. Specifically, we generated a mutant with a D → N change in the conserved DTG motif and, only for the prokaryotic expression, another mutant with a R → K change in the GRDLL motif. Both mutants were previously shown to render HML-2 and HIV-1 protease inactive [44, 74]. Plasmid constructs were verified by Sanger sequencing.

### Plasmids for eukaryotic expression of epitope-tagged human cellular proteins presumably processed by HERV-K(HML-2) pro

Full-length coding sequences for HSP90AA1, CIAPIN1, C15orf57, MAP2K2 and TUBA1A were obtained from GE Healthcare/Dharmacon and cloned into pcDNA3 with a human influenza hemagglutinin (HA) tag added during the cloning procedure. To do so, each full-length ORF was amplified by PCR. The forward PCR primer was the same as the one used for generation of PCR products for in vitro translation of proteins (see above). The reverse PCR primer added an HA-tag in frame at the ORF’s 3′ end. The PCR product was cloned into pGEM T-Easy, released by a *Not*I digest and cloned into *Not*I-digested pcDNA3 vector. Clones were verified by Sanger sequencing.

Other protein cDNAs of interest were cloned into pEZYflag (Addgene Plasmid #18700) [75] using LR Clonase II and Gateway technology, and contained an N-terminal FLAG-epitope tag. Those cloned coding sequences were from Ultimate ORF libraries (Thermo Fisher Scientific/Invitrogen) made available through the ChemCORE facility at Johns Hopkins University, Baltimore MD, USA (see Additional file 1: Table S5 for clone identifiers). Other coding sequences for proteins of interest were obtained from Addgene: pcDNA3 HA eIF4GI (1–1599) (plasmid #45640) [76], and pCMV-Tag2B EDD FLAG-UBR5 (plasmid #37188) [77]. Dr. V. Dawson, Johns Hopkins University School of Medicine, kindly provided Myc-STUB1 (CHIP) [78]. HSPA5 cDNA was obtained from Dr. D.L. George, University of Pennsylavania, and was recloned by PCR in the vector pcDNA6 myc/his B (Invitrogen) with a C-terminal T7-tag.

### Cell culture

Human embryonic kidney (HEK) 293 T cells (ATCC, or The Leibniz Institute DSMZ – German Collection of Microorganisms and Cell Cultures), human osteosarcoma U2OS cells (a gift from Dr. N. Kedersha, Harvard University), and human cervical cancer HeLa cells (DSMZ) were grown in Dulbecco’s modified Eagle’s medium (DMEM), supplemented with 10% heat inactivated fetal bovine serum (Sigma-Aldrich, or Merck-Millipore), GlutaMax, and Pen-Strep (Invitrogen/FisherScientific).

### Prokaryotic expression and purification of HERV-K(HML-2.HOM) protease

Expression and purification of HML-2 Pro followed a previously described protocol [44] with minor modifications. In brief, *Escherichia coli* BL21(DE3) cells harboring plasmid pET11dPro (see above) were inoculated into 100 ml Luria-Bertani (LB_Amp_) medium supplemented with ampicillin (100 μg/ml) and incubated overnight at 37 °C. 20 ml of the overnight culture were then inoculated into 1 L of LB_Amp_ medium and incubated at 37 °C until A_600_ = 0.6 was reached. Expression of HML-2 Pro was induced by addition of isopropyl-1-thio-β-D-galactopyranoside (Sigma) at a final concentration of 0.4 mM. After 3 h at 37 °C bacterial cells were pelleted by centrifugation at 6800 g for 30 min at 4 °C. Cells were resuspended in 50 ml of pre-cooled 5× TE buffer (0.1 M Tris/HCl, 5 mM EDTA, pH 7.5) and subjected to sonication (10 × 10 s, 40 W) on ice. The cell lysate was centrifuged for 30 min at 3600 g and 4 °C and the soluble fraction was discarded. Inclusion bodies were washed twice with 20 ml of 5× TE buffer and then dissolved in 100 ml of 8 M urea, 0.1 M Tris/HCl pH 7.5, 1 mM DTT. Refolding of HML-2 Pro was achieved by dialyzing the solution against 4 L of 20 mM PIPES, pH 6.5, 1 M NaCl, 1 mM DTT, at 4 °C for 3 h and then against 4 L of fresh buffer overnight. During renaturation, the HML-2 Pro precursor of 18 kDa completely autoprocessed into the mature 12 kDa form. The solution was centrifuged for 30 min at 6800 g and 4 °C to eliminate precipitated proteins and then mixed 1:1 with buffer A (50 mM PIPES, pH 6.5, 1 M NaCl, 1 mM EDTA, 1 mM NaK tartrate, 10% [v/v] glycerol). Five ml of Pepstatin A-agarose (Sigma), pre-washed in H_2_O_dd_ and then buffer A, were added and the mixture was incubated overnight at 4 °C with slow agitation and subsequently packed onto a chromatography column pre-conditioned with Buffer A. Aliquots from flow-through and two fractions from wash steps were collected (see below). Bound proteins were eluted by gravity with Buffer B (0.1 M Tris/HCl, pH 8.0, 1 mM NaK tartrate, 10% [v/v] glycerol, 5% [v/v] ethylene glycol) collecting 6 elution fractions of 5 ml each. The various purification steps were monitored by SDS-PAGE followed by Coomassie-staining of PAA-gels. Protease-containing elution fractions were pooled and concentrated using an Amicon centrifugal filter (3000 MWCO) to a final volume of about 2 ml. Protease concentration was determined by DC Protein assay (Biorad) and UV spectrophotometry using a calculated molar absorption coefficient of 29,115 M^− 1^ cm^− 1^ of expressed HML-2 Pro. The protein solution was aliquoted and stored at − 80 °C.

### Optimization of HERV-K(HML-2.HOM) protease activity by in vitro enzymatic fluorescence assays

We monitored and optimized HERV-K(HML-2.HOM) Pro activity by varying buffer composition, pH, and Pepstatin A concentration. A synthetic fluorescent Anthranilyl-Substrate Trifluoroacetate salt, 2-aminobenzoyl-Thr-Ile-Nle-p-nitro-Phe-Gln-Arg-NH_2_ (4,030,748, BACHEM), a known substrate of HIV Pro [46], was dissolved in DMSO to produce a 1.06 mM stock solution. Purified HERV-K(HML-2.HOM) mature Pro was diluted in Buffer B to a final concentration of 4.6 μM (see above). Protease was then incubated at a final concentration of 460 nM with fluorescent substrate (final concentration 20 μM, 40 μM for Pepstatin A inhibition experiments) in a final volume of 50 μl. Reactions were monitored at 37 °C by detecting increase in fluorescent signal for each reaction condition every 4 min for up to 180 min. Fluorescence measurements were taken in 96-well microplates (Greiner Bio-One 655,087) using a Tecan Infinite m200 spectrophotometer with excitation at 280 nm and emission measured at 420 nm. The amount of fluorescent product, thus HML-2 Pro activity, was calculated based on changes in fluorescence emission.

### Preparation of Hela total cell lysate

Human cervical adenocarcinoma (HeLa) cells were cultured at 37 °C and 5% [v/v] CO_2_ in Dulbecco’s Modified Eagle’s Medium supplemented with 10% [v/v] heat inactivated fetal calf serum, 50 μg/ml penicillin, and 50 μg/ml streptomycin. A total of 1.4•10^8^ cells grown to near confluence in eight 160 cm^2^ tissue culture flasks were washed with 1× PBS and detached by trypsinization. Cells were collected in 20 ml 1× PBS, pelleted for 5 min at 250 g, resuspended in 0.5 ml of 5 mM MES, pH 6.0 supplemented with protease inhibitors (cOmplete,Mini, EDTA-free, Roche) at the recommended concentration, and subjected to lysis by three freeze-thaw cycles. The protein lysate was centrifuged at 4 °C for 30 min. at 16,100 g. The supernatant was stored in aliquots at − 80 °C. Protein concentration was measured using the Biorad DC Protein Assay Kit.

### Incubation of HeLa total cell lysate with purified HERV-K(HML-2) protease and subsequent TAILS analysis

In a total reaction volume of 2 ml, we incubated 2 mg of HeLa proteins with purified HML-2 Pro (200 nM final concentration) in a buffer composed of 0.1 M PIPES, 1 M NaCl, and 2% [v/v] DMSO, pH 5.5 or pH 7. Two replicates were performed. Additional control reactions for each condition contained Pepstatin A at 200 μM that was concluded to effectively inhibit HML-2 Pro activity. All reactions were incubated for 75 min. at 37 °C and stored at − 80 °C until TAILS analysis (see below). TAILS was performed essentially as described previously [42, 43], comparing HML-2 Pro-treated HeLa total cell lysate to control reactions for the two replicates performed at pH 5.5 and at pH 7. An Easy-LC 1000 coupled to a Q-Exactive plus mass spectrometer was used for LC-MS analysis. The mass spectrometry proteomics data have been deposited to the ProteomeXchange Consortium via the PRIDE [79] partner repository (dataset identifiers PXD010159 and PXD013296).

### In vitro translation of proteins presumably processed by HERV-K(HML-2) protease

The coding region of full-length protein was PCR-amplified from purified plasmid template DNA (see above). Forward primers were located at the start codon and included a 5′ extension consisting of a *Bam*HI restriction site, a T7 promoter, a spacer and a Kozak consensus sequence for translation-initiation (5′-GGATCC|TAATACGACTCACTATAGGG|AACAG|CCACCATG [cDNA candidate protein]-3′). The reverse primers added sequence encoding a human influenza hemagglutinin (HA) epitope tag and a stop codon (5′-TTA|AGCGTAATCTGGAACATCGTATGGGTA[cDNA candidate protein]-3′) at the end to the PCR product’s protein coding sequence. The standard PCR mix contained primers at a final concentration of 0.25 μM, 100 μM dNTP mix, 2.5 U Taq polymerase (Sigma), and 5 ng template DNA in a final reaction volume of 50 μl. PCR cycling conditions were as follows: 3 min. at 94 °C; 30 cycles of 50 s. at 94 °C, 50 s. at 56 °C, 3 min at 72 °C; and a final 10 min. at 72 °C. PCR products directly served as template using a TnT T7 Quick Coupled Transcription/Translation System (Promega) following the manufacturer’s recommendations. Briefly, 2.5 μl of a PCR reaction were added to 22 μl of TNT T7 PCR Quick Master Mix containing either 0.5 μl of HPLC-purified, translation-grade L-^35^S-methionine (370 MBq, 10 mCi/ml; Hartmann Analytic, Braunschweig, Germany) or 0.5 μl of 1 mM “cold” methionine, incubated for 90 min. at 30 °C and frozen at − 20 °C immediately afterwards.

### Incubation of candidate proteins with purified HERV-K(HML-2) protease in vitro

In vitro transcribed/translated radioactively or HA-tag-labeled candidate protein was incubated with purified HML-2 Pro to potentially confirm in vitro processing by HML-2 Pro. Briefly, 1 μl of the TNT® T7 in vitro transcription/translation reaction was incubated with 400 nM purified HML-2 Pro in a buffer of 1 M NaCl, and 0.1 M PIPES pH 5.5, for 180 min. at 37 °C in a final volume of 16 μl. Control reactions included Pepstatin A at 400 μM. The entire reaction was subjected to SDS-PAGE (see below).

### Co-expression of candidate proteins and HERV-K(HML-2) protease in HEK293T cells

In the case of Western blots shown in Fig. 6a, b (top) and Additional file 2: Figure S3 (those with Coomassie staining), HEK293T cells were seeded at a density of 2•10^5^ cells per well in a 12-well plate. The following day, cells were transfected with either phCMV-Pro-wt, phCMV-Pro-mut and phCMV, or pEGFP-Pro-wt, pEGFP-Pro-mut and pEGFP. Candidate protein cDNAs (HSP90AA1, CIAPIN1, C15orf57, MAP2K2, TUBA1A) cloned in pcDNA3 vector were co-transfected. Transfections were performed using Fugene HD Transfection Reagent (Promega) at a DNA:Fugene ratio of 1:3. Each plasmid combination was transfected in duplicate in two different wells. Combinations of plasmids were co-transfected as follows. EGFP-fused wild-type protease + candidate protein: 0.5 μg pEGFP-Pro-wt + 0.5 μg pcDNA3-candidate; EGFP-fused mutated protease + candidate protein: 0.5 μg pEGFP-Pro-mut + 0.5 μg pcDNA3-candidate; GFP-Control: 0.5 μg pEGFP + 0.5 μg pcDNA3-candidate; wild-type protease + candidate protein: 0.5 μg phCMV-Pro-wt + 0.5 μg pcDNA3-candidate; mutated Pro + candidate protein: 0.5 μg phCMV-Pro-mut + 0.5 μg pcDNA3-candidate; Control: 0.5 μg phCMV + 0.5 μg pcDNA3-candidate. 24 h post transfection, ~ 0,8•10^6^ cells each were washed with 1xPBS, trypsinized and pelleted by centrifugation for 5 min. at 300 g in 1xPBS. Cell pellets were resuspended and lysed in 100 μl of RIPA buffer (150 mM NaCl, 1% [v/v] NP40, 0.5% [w/v] sodium deoxycholate, 0,1% [w/v] SDS, 50 mM Tris-HCl pH 8.0 and 5 mM EDTA), supplemented with protease inhibitors (cOmplete Mini, EDTA-free, Roche) and Pepstatin-A (Merck Chemicals) at 1 μg/μl final concentration. Insoluble cell debris was pelleted by centrifugation at ~ 13,000 g for 15 min. at 4 °C. Protein concentration was measured using the DC Protein Assay Kit (Biorad).

In the case of Western blots shown in Fig. 6b (bottom) and Additional file 2: Figure S3 (those with Ponceau S staining), HEK293T cells seeded in 6-well plates were co-transfected with test plasmids together with pcDNA6 myc/his B or pcDNA5 FRT/TO empty vector (Invitrogen/Thermo Fisher Scientific), phCMV-Pro-wt, or phCMV-Pro-mut. After 18 h, MG132 (Millipore-Sigma) at a final concentration of 10 μM was added and cells were incubated for an additional 4–5 h. Cells from duplicate wells were pooled and lysed with RIPA Buffer (Millipore-Sigma) supplemented with Mammalian Protease Inhibitor Cocktail (Sigma) and 2 mM phenylmethylsulfonyl fluoride (PMSF). Note that Protease Inhibitor Cocktail contains Pepstatin A. Protein lysates were sonicated with a Diagenode Bioruptor and centrifuged at 13000 g at 4 °C for 15 min to recover supernatant. Protein concentrations were determined with the Pierce BCA Protein Assay Kit (Thermo Fisher Scientific).

### SDS-PAGE and detection of labeled proteins

In the case of Western blots shown in Fig. 6a, b (top) and Additional file 2: Figure S3 (those with Coomassie staining), between 15 and 20 μg of each total protein sample, with equal amounts of each protein sample loaded per candidate protein examined, were subjected to reducing SDS-PAGE using a Bis-Tris buffer system. Protein lysates were mixed with 4× NuPAGE LDS Sample Buffer (Thermo Fisher Scientific) and DTT at 50 mM final concentration, denatured for 15 min. at 65 °C, and briefly centrifuged. Protein samples were loaded and separated in 10% or 12% Bis-Tris polyacrylamide gels at 180 V in XCell SureLock™ Mini-Cells using NuPAGE MES SDS or MOPS SDS Running Buffer and optional NuPAGE Antioxidant.

Polyacrylamide gels with radiolabeled proteins were fixed for 30 min. in 50% [v/v] methanol/10% [v/v] acetic acid, then soaked in distilled water three times for 10 min each. Gels were dried for 2 h at 80 °C under vacuum and subsequently exposed to a Storage Phosphor screen (Amersham Biosciences) at room temperature for 16 h. The screen was scanned using a Typhoon 9410 scanner (GE Healthcare).

Detection of cold proteins was done by Western blot. Following SDS-PAGE, proteins were transferred onto Hybond 0.2 μm PVDF membrane (Amersham/GE Healthcare) using a XCell II™ Blot module and NuPAGE Transfer Buffer in the presence of NuPAGE Antioxidant. Blot membranes were blocked in 1× TBS, 5% [w/v] nonfat dry milk for 1 h and incubated overnight at 4 °C with an α-HA rat monoclonal antibody diluted 1:500 in 1× TBS/5% [w/v] nonfat dry milk. Detection of proteins of interest employed antibodies specific for HA-tag, EGFP, and HML-2 Pro [19]. Secondary antibody incubation was done using peroxidase-coupled rabbit α-rat IgG (Sigma-Aldrich; A5795) or goat α-rabbit IgG (Sigma-Aldrich; A0545) each diluted at 1:5000, for 2 h at room temperature. α-HA rat monoclonal (clone 3F10) and rabbit α-rat antibodies were generously provided by Friedrich Grässer, Institute of Virology, University of Saarland. Signal detection was performed using SignalFire™ Elite ECL Reagent (Cell Signaling Technology) and Chemidoc™ Imaging System (Bio-Rad). Image analysis utilized ImageLab 5.2.1 software (Bio-Rad). Loading of equal protein amounts was verified by staining blot membranes with Coomassie Brilliant Blue after the ECL procedure.

In the case of Western blots shown in Fig. 6b (bottom) and Additional file 2: Figure S3 (those with PonceauS staining), 35 μg of total protein resuspended in 3× SDS loading buffer (187.5 mM Tris-HCl (pH 7.5), 6% [w/v] SDS, 30% [v/v] glycerol, 0.03% [w/v] bromophenol blue, 2% [v/v] β-mercaptoethanol) was subjected to reducing SDS-PAGE in XCell SureLock™ Mini-Cells with 4–12% NuPAGE Bis-Tris polyacrylamide gels and MOPS SDS Running Buffer. Proteins were blotted as above, except, PVDF membranes were blocked in 1× PBS, 5% [w/v] nonfat dry milk for 1 h and incubated overnight at 4 °C with primary antibodies for epitope tags or endogenous proteins in 1× PBS/2.5% [w/v] nonfat dry milk/0.5% Tween 20. Antibodies used included mouse α-T7-Tag (Novagen, diluted 1:4000), and rabbit α-DYKDDDDK (FLAG)-tag (clone D6W5B), mouse α-HA-tag (clone 6E2), rabbit α-Myc-tag (clone 71D10), and rabbit α-HSP90 (clone C45G5) (all from Cell Signaling Technology, diluted 1:1500), and donkey horseradish peroxidase-conjugated secondary antibodies from Jackson ImmunoResearch Laboratories (diluted 1:10,000). Signal detection was performed using SuperSignal West Pico PLUS Chemiluminescent Substrate (Thermo Fisher Scientific) and Hyperfilm ECL (Sigma Aldrich). Loading of equal protein amounts was verified by Ponceau S staining of membranes after the ECL procedure.

### Identification of HERV-K(HML-2) loci potentially encoding protease

Reference and non-reference HERV-K(HML-2) locus sequences were analyzed for presence of pro ORFs. HML-2 pro is translated via a ribosomal frameshift between the HML-2 gag and pro ORFs. We therefore also analyzed for presence of a gag ORF in respective HML-2 sequences. Pro ORFs of HML-2 loci fulfilling criteria were translated in silico, multiply aligned, and further analyzed for presence of catalytic motifs conserved in retroviral aspartate proteases

## Additional files

Additional file 1: **Tables S1a–d.** Results of TAILS analyses at pH 5.5 and pH 7 showing replicates 1 and 2 for each. **Table S2.** Selected human proteins with multiple cleavages by HERV-K(HML-2) protease. **Table S3.** Selected information from the Catalogue of Somatic Mutations in Cancer (COSMIC) for human genes for which encoded proteins were identified as substrates of HML-2 Pro. **Table S4.** Selected information from the Online Mendelian Inheritance in Man (OMIM) database for human genes for which encoded proteins were identified as substrates of HML-2 Pro. **Table S5.** Clone identifiers of cloned coding sequences of proteins investigated for processing by HERV-K(HML-2) Protease. (XLSX 1290 kb)

Additional file 2: **Figure S1.** Self-processing of HERV-K(HML-2) Protease during purification. **Figure S2.** Additional examples of verifications of processing of human proteins by HERV-K(HML-2) Protease in vitro. **Figure S3.** Additional examples of verifications of processing of human proteins by HERV-K(HML-2) Protease in vivo and documentation of loading controls. **Figure S4.** Quantification of GFP-positive live cells and exclusion of processed protein products due to caspase activity. **Figure S5.** Localization of EGFP-Pro-mut in human osteosarcoma U2OS and HEK293T cells. **Figure S6.** Evidence for presence of HERV-K(HML-2) Protease in cell lines known to express HERV-K(HML-2) at relatively high levels. (PDF 5270 kb)

## Data Availability

The mass spectrometry proteomics data have been deposited at the ProteomeXchange Consortium via the PRIDE partner repository (dataset identifiers PXD010159 and PXD013296).

## Abbreviations

aa: amino acids
DMSO: dimethyl sulfoxide
EGFP: enhanced green fluorescent protein
GO: gene ontology
h: hours
HERV: human endogenous retrovirus
HIV: human immunodeficiency virus
HML: human MMTV-like
kDa: kilodalton
MES: 2-(N-morpholino)ethanesulfonic acid
MOPS: 3-(N-morpholino)propanesulfonic acid
np9: novel protein of 9 kDa
nt: nucleotide
PAA: polyacrylamide
PCR: polymerase chain reaction
PIPES: piperazine-N,N′-bis(2-ethanesulfonic acid)
Pro: protease
rec: regulator of expression encoded by corf
TAILS: Terminal Amine Isotopic Labeling of Substrates
