# Supplementary material for: A human endogenous retrovirus encoded protease potentially cleaves numerous cellular proteins
Source: Mob DNA. 2019 Aug 22;10:36. doi: 10.1186/s13100-019-0178-z (PMC6707001; doi:10.1186/s13100-019-0178-z)
Supplement: Supplementary file 2 — Figure S1. Self-processing of HERV-K(HML-2) Protease during purification. Figure S2. Additional examples of verifications of processing of human proteins by HERV-K(HML-2) Protease in vitro. Figure S3. Additional examples of verifications of processing of human proteins by HERV-K(HML-2) Protease in vivo and documentation of loading controls. Figure S4. Quantification of GFP-positive live cells and exclusion of processed protein products due to caspase activity. Figure S5. Localization of EGFP-Pro-mut in human osteosarcoma U2OS and HEK293T cells. Figure S6. Evidence for presence of HERV-K(HML-2) Protease in cell lines known to express HERV-K(HML-2) at relatively high levels. (PDF 5270 kb) [file 13100_2019_178_MOESM2_ESM.pdf]

A human endogenous retrovirus encoded protease potentially cleaves numerous cellular proteins

Giuseppe Rigogliuso[1], Martin L. Biniossek[2], John L. Goodier[3], Bettina Mayer[2], Gavin C. Pereira[3], Oliver Schilling[4,5], Eckart Meese[1], Jens Mayer[1]

[1] Department of Human Genetics, Medical Faculty, University of Saarland, Homburg, Germany
[2] Institute of Molecular Medicine and Cell Research, University of Freiburg, Freiburg, Germany
[3] McKusick-Nathans Institute of Genetic Medicine, Johns Hopkins University School of Medicine, Baltimore, Maryland, USA
[4] Institute of Surgical Pathology, Medical Center, University of Freiburg, Freiburg, Germany
[5] German Cancer Consortium (DKTK) and German Cancer Research Center (DKFZ), Heidelberg, Germany

Additional file 2: Supplemental Figures

Figure S1. Self-processing of HERV-K(HML-2) Protease during purification

Figure S2. Additional examples of verifications of processing of human proteins by HERV-K(HML-2) Protease *in vitro*

Figure S3. Additional examples of verifications of processing of human proteins by HERV-K(HML-2) Protease in vivo and documentation of loading controls

Figure S4. Quantification of GFP-positive live cells and exclusion of processed protein products due to caspase activity

Figure S5. Localization of EGFP-Pro-mut in human osteosarcoma U2OS and HEK293T cells

Figure S6. Evidence for presence of HERV-K(HML-2) Protease in cell lines known to express HERV-K(HML-2) at relatively high levels

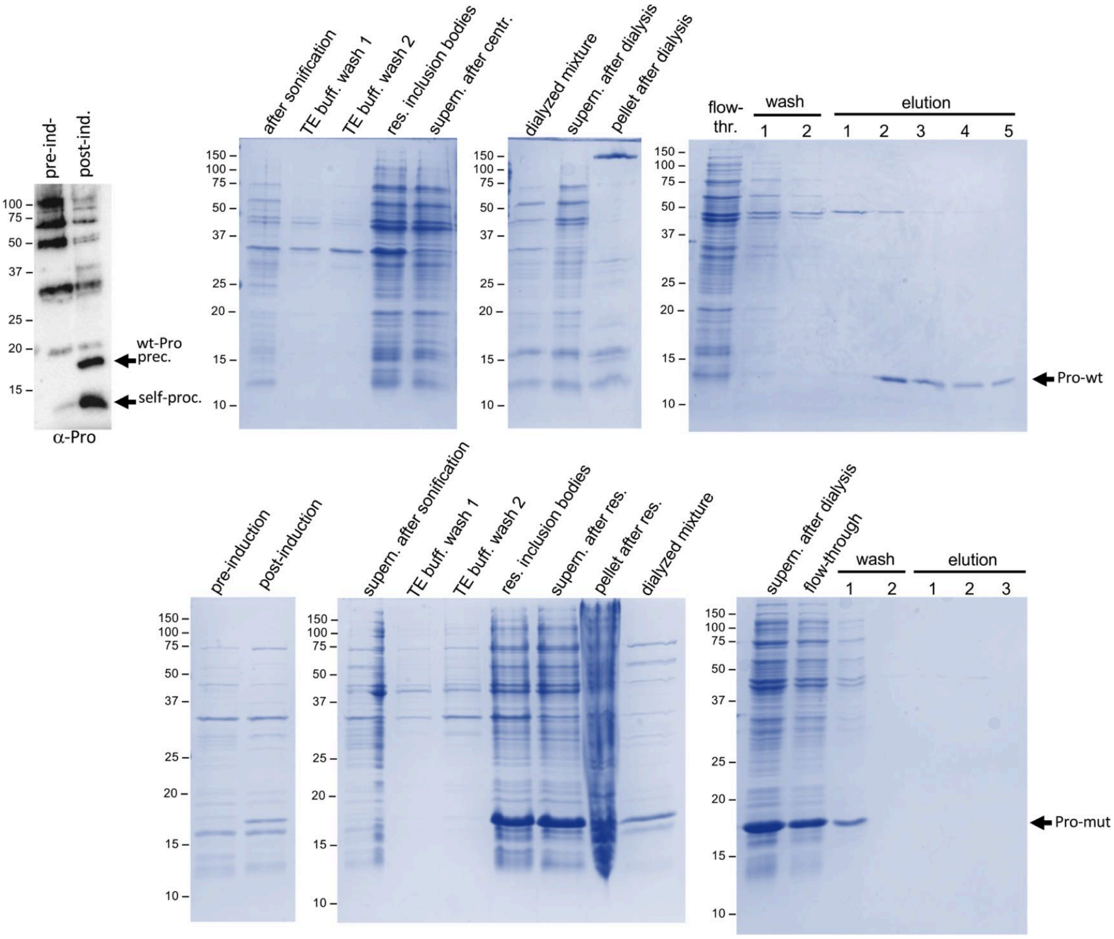

Figure S1. Self-processing of HERV-K(HML-2) Protease during purification.
A previously established method was employed for purification of prokaryotically expressed HML-2 wild-type Pro (top) and a Pro mutated for the DTG motif (bottom). See the main paper text for details. Samples were taken at various steps of the procedure: cell lysate after sonification (supernatant after sonification and centrifugation for the bottom panel); two washes of inclusion bodies in TE buffer; resuspension of inclusion bodies in a urea-containing buffer; supernatant (supern.) (and pellet for the bottom panel) after centrifugation of resuspended (res.) inclusion bodies; protein mixture after dialysis; supernatant and pellet (for the top panel) after dialysis and centrifugation; flow through ("flow-thr.") after incubation of dialyzed protein mixture with Pepstatin A-agarose; two washes after binding; five elution fractions. Proteins in samples were separated by SDS-PAGE. PAA-gels were stained with Coomassie. Molecular masses of marker proteins are reported. Note that prokaryotically expressed HML-2 wild-type Pro self-processed during the purification/renaturation procedure giving rise to an approximately 12 kDa protein (arrow in top panel). Further note that the approximately 18 kDa precursor of wt-Pro already self-processes to the 12 kDa mature form during prokaryotic expression in BL21(DE3) cells, as demonstrated by Western blot analysis of samples taken before and after induction of Pro-expression and using an α-Pro polyclonal antibody (top panel, left; see also the main paper text). Also note that mutant Pro, which cannot be purified efficiently, did not self-process and remained as a precursor of approximately 18 kDa. A Coomassie-stained gel showing presence of mutant-Pro precursor after induction is shown in the bottom panel, left.

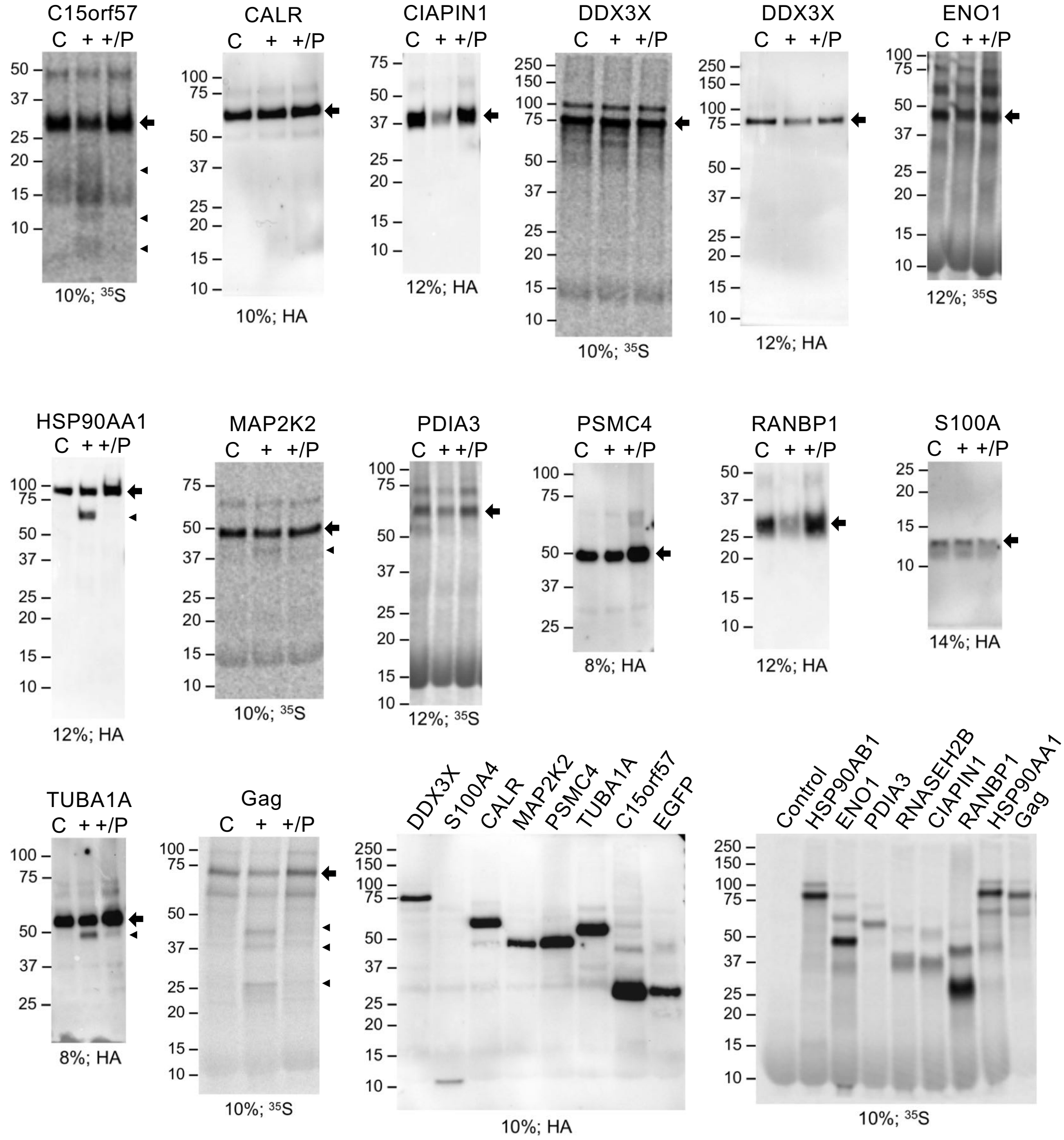

Figure S2. Additional examples of verifications of processing of human proteins by HERV-K(HML-2) Protease *in vitro*.

Human candidate proteins were expressed *in vitro* using a coupled transcription/translation system. Results from protease incubations of various candidate proteins labeled with either ^{35}S-methionine or a C-terminal HA-tag ("^{35}S" or "HA") are shown. Experiments included for each candidate protein a reaction without protease ("C"), one with protease ("+"), and one with protease and pepstatin A ("+/P"). Reaction products were separated by SDS-PAGE in PAA-gels (acrylamide concentrations as indicated) and processed for phosphorimager analysis or HA-tag-specific Western blots depending on the label. Processing of full-length candidate proteins (indicated by an arrow) was evidenced by additional protein bands smaller than the respective full-length candidate protein (indicated by arrowheads, if visible), and/or a decrease in the amount of full-length candidate protein (see the Results section of the main paper text). *In vitro* translated full-length proteins, detected via HA-tag or the ^{35}S-label, are shown in the lower right. EGFP and HERV-K(HML-2) Gag protein served as controls for translation efficiency. Incubation of HERV-K(HML-2) Gag with HML-2 Pro, depicted in the bottom row, served as an additional control.

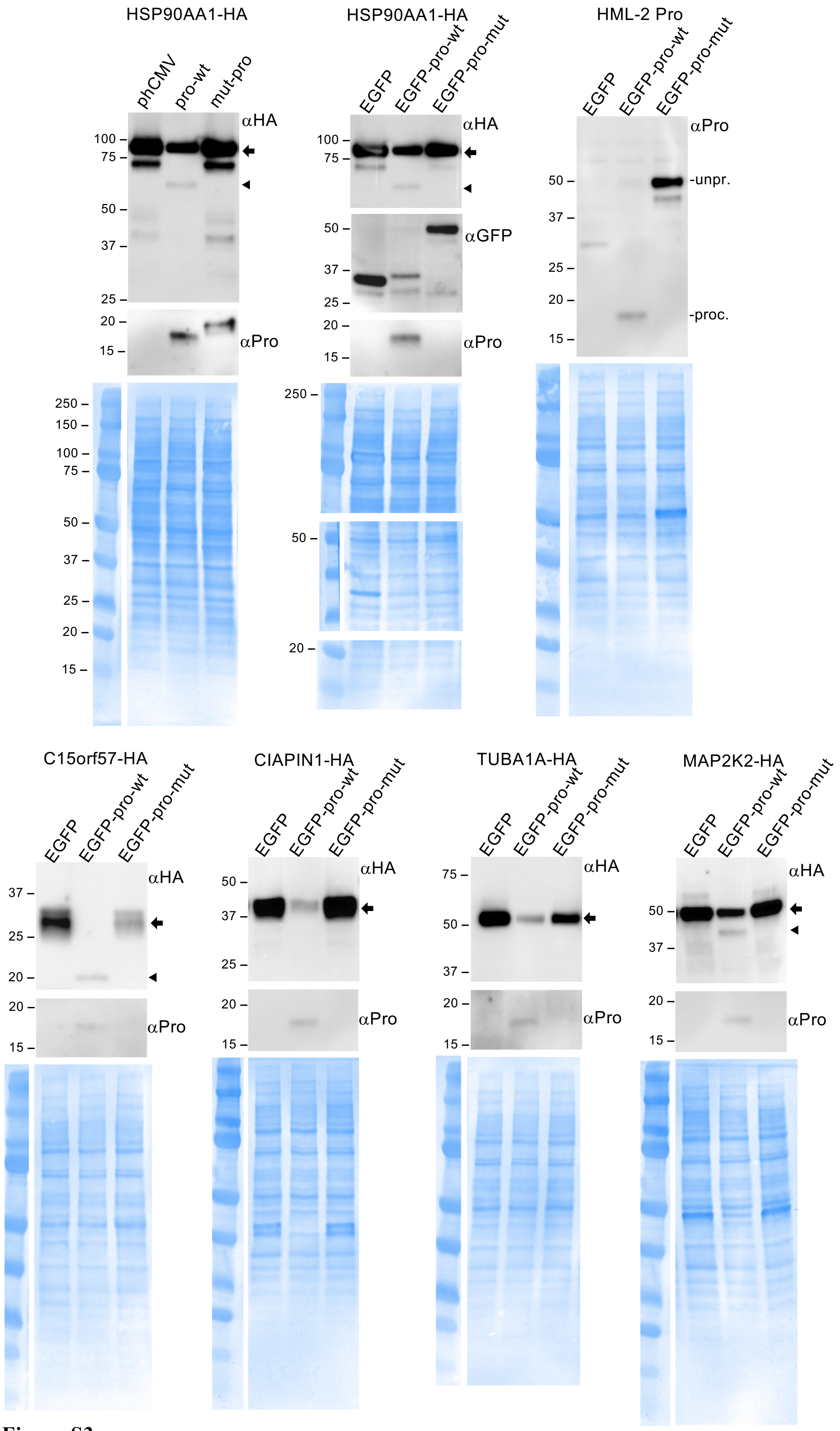
HSP90AA1-HA
phCMV
pro-wt
mut-pro
αHA
αPro
HSP90AA1-HA
EGFP
EGFP-pro-wt
EGFP-pro-mut
αGFP
HML-2 Pro
-unpr.
-proc.
C15orf57-HA
CIAPIN1-HA
TUBA1A-HA
MAP2K2-HA

Figure S3

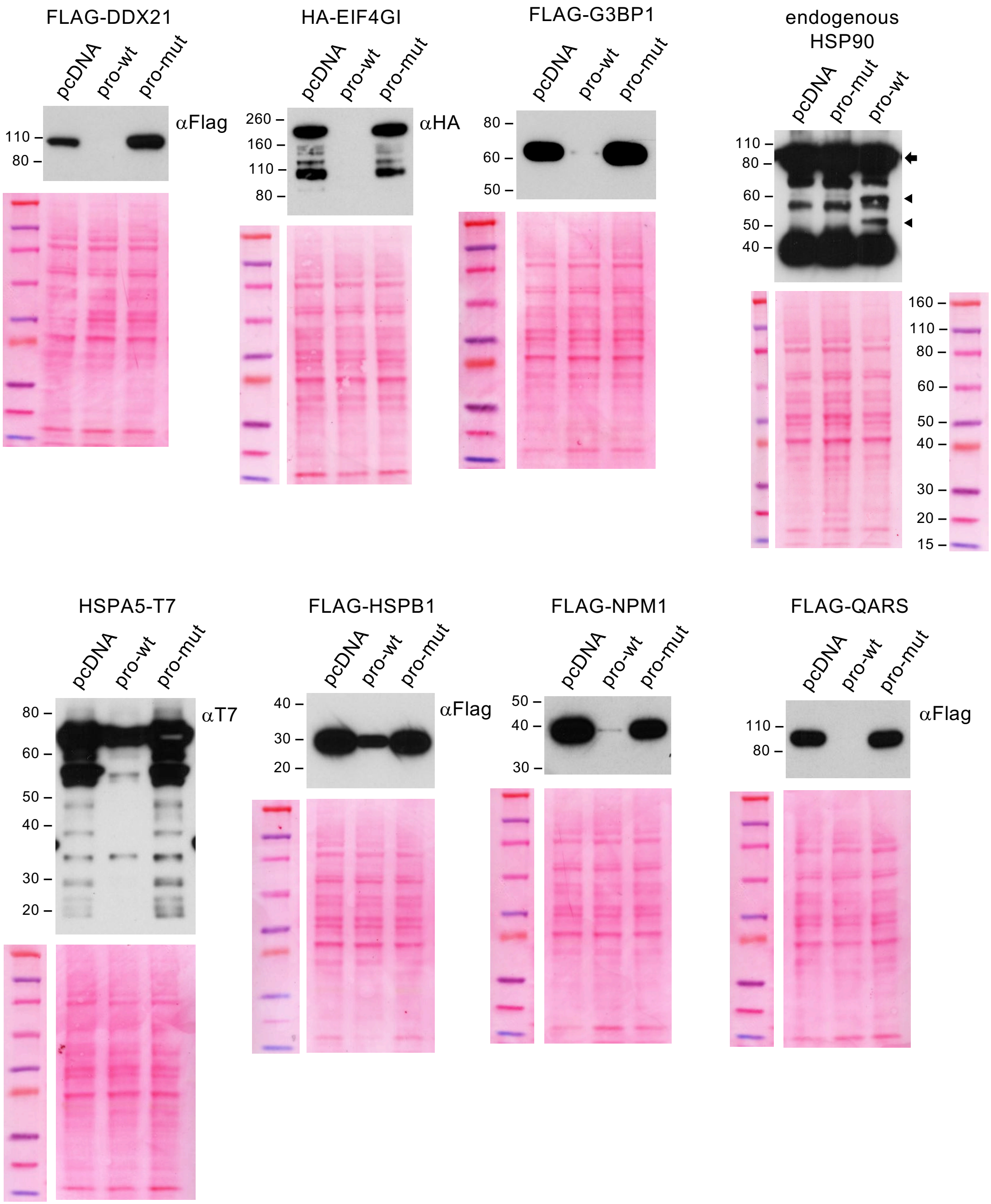

Figure S3 – cont.

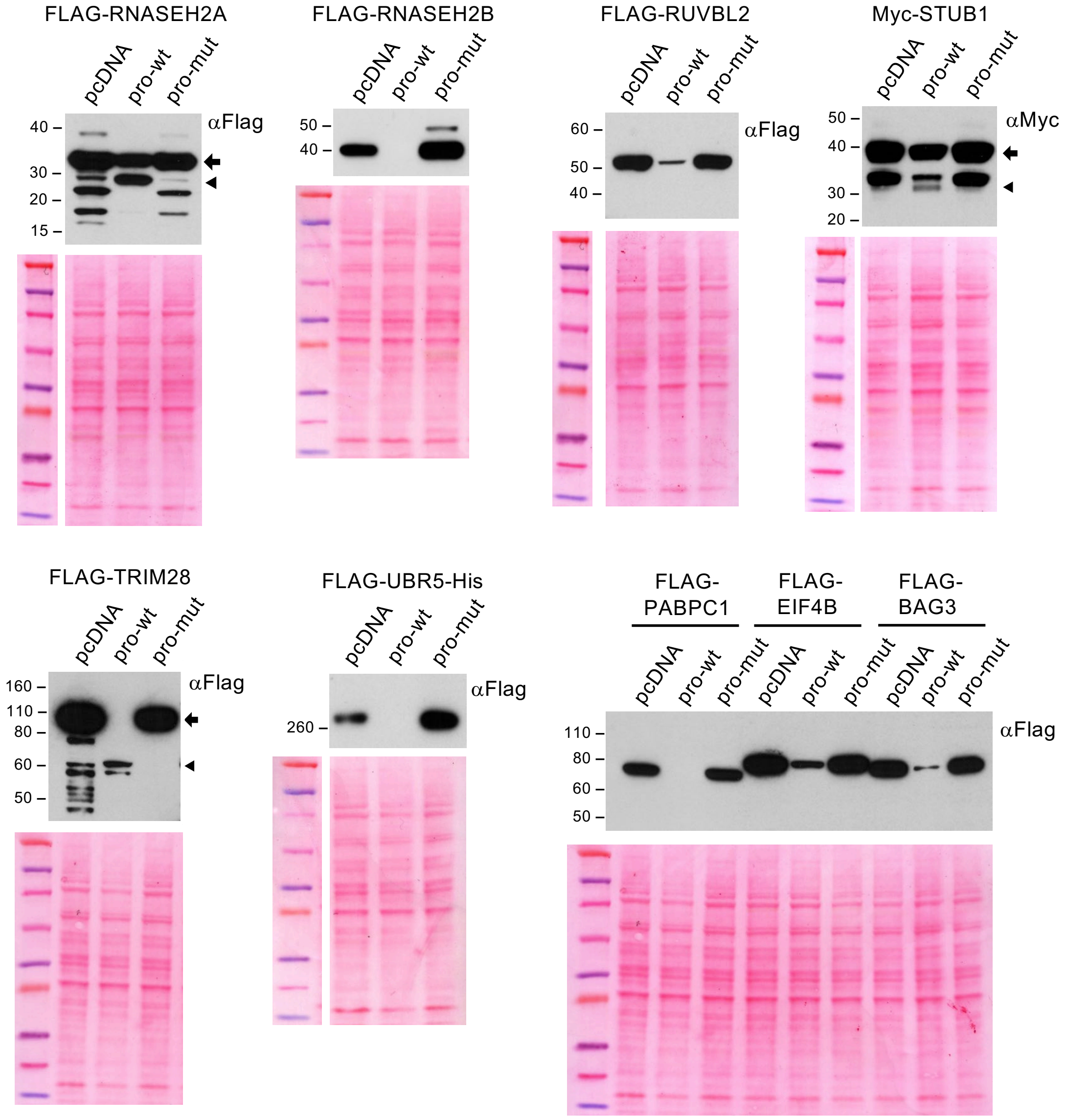

Figure S3. Additional examples of verifications of processing of human proteins by HERV-K(HML-2) Protease *in vivo* and documentation of loading controls. Western blot results presented in the main paper are shown again for the sake of convenience. Furthermore, results for human candidate proteins such as BAG3, DDX21, EIF4B, EIF4G1, G3BP1, HSPA5, HSPB1, NPM1, PABPC1, QARS, RNASEH2B, RUVBL2, and UBR5 are shown. After the ECL procedure, Western blot membranes were stained with either Coomassie Brilliant Blue or Ponceau S. Respective membranes are shown each. Sizes of marker proteins are indicated. Precision Plus Protein™ Dual Color Standard (Bio-Rad) and Novex™ Sharp Prestained Protein Standard (Fisher Scientific; see separate panel for marker molecular weights) were used for experiments with Coomassie or Ponceau S-stained membranes, respectively. See the main paper for more details on experimental procedures.

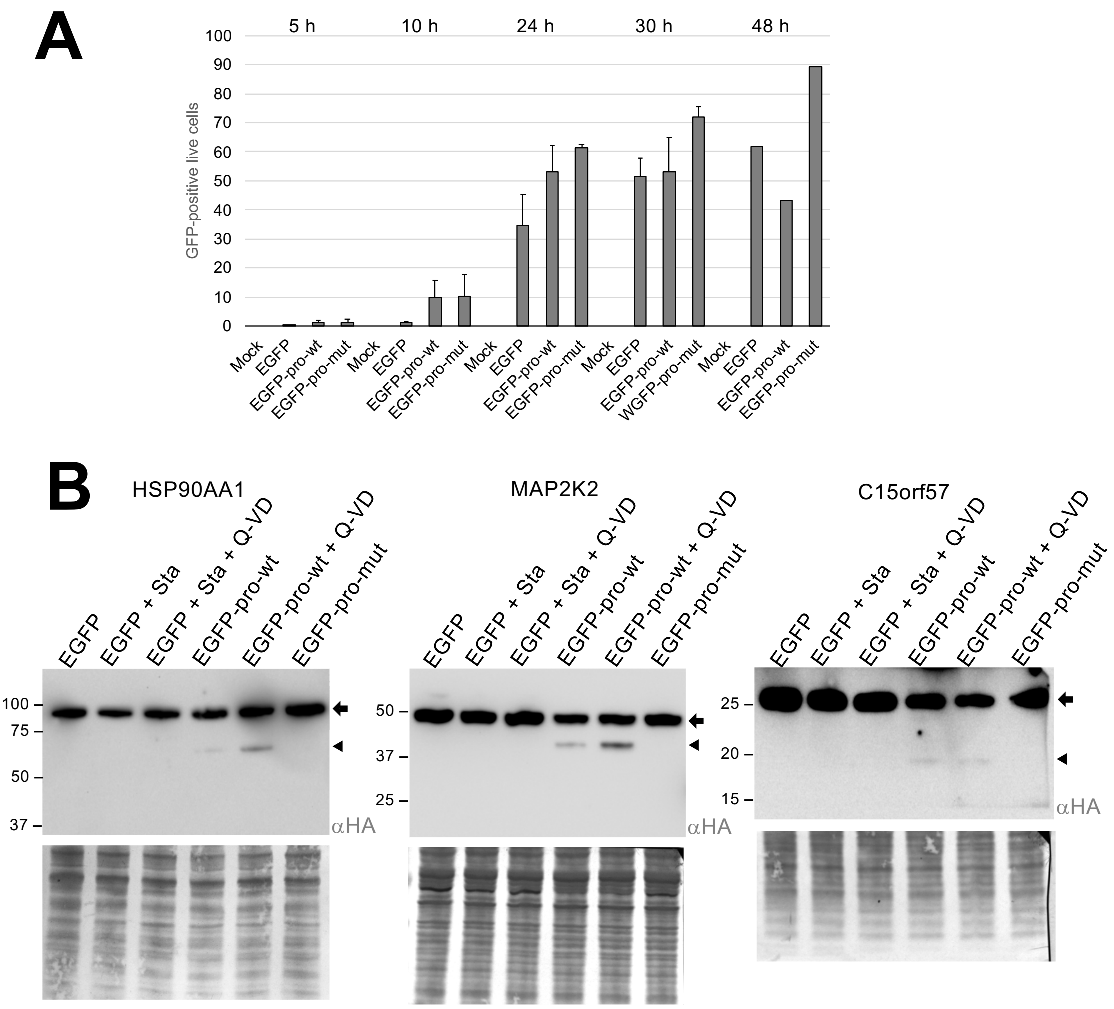

Figure S4. Quantification of GFP-positive live cells and exclusion of processed protein products due to caspase activity. A. HEK293T cells were transiently transfected with plasmids encoding GFP, GFP-Pro-wt, or GFP-Pro-mut. Relative numbers of GFP-positive cells were quantified by FACS analysis after the indicated time periods and are expressed as percentage of total number of cells analyzed each. Means and standard deviations from two different experiments are given. Between 9000 and 150,000 cells were gated. **B.** Selected human proteins co-expressed with HML-2 Pro and induction of apoptosis. Indicated HA-tagged human proteins were transiently co-expressed with GFP, GFP-Pro-wt or GFP-Pro-mut in HEK293 cells. Some reactions harbored, in addition, pan-caspase inhibitor (Q-VD; 25 μM). Apoptosis was induced by addition of Staurosporin at 2 μM and total cell lysate was prepared 5 h later. Following Western blotting, candidate proteins were detected using an anti-HA antibody. Note that Staurosporin did not result in additional protein bands smaller in size than full-length candidate proteins, as seen in reactions expressing HML-2 Pro-wt. This lends further support to those smaller protein bands being products of candidate proteins processed by HML-2 Pro rather than products from caspase activity following HML-2 Pro-induced cell death.

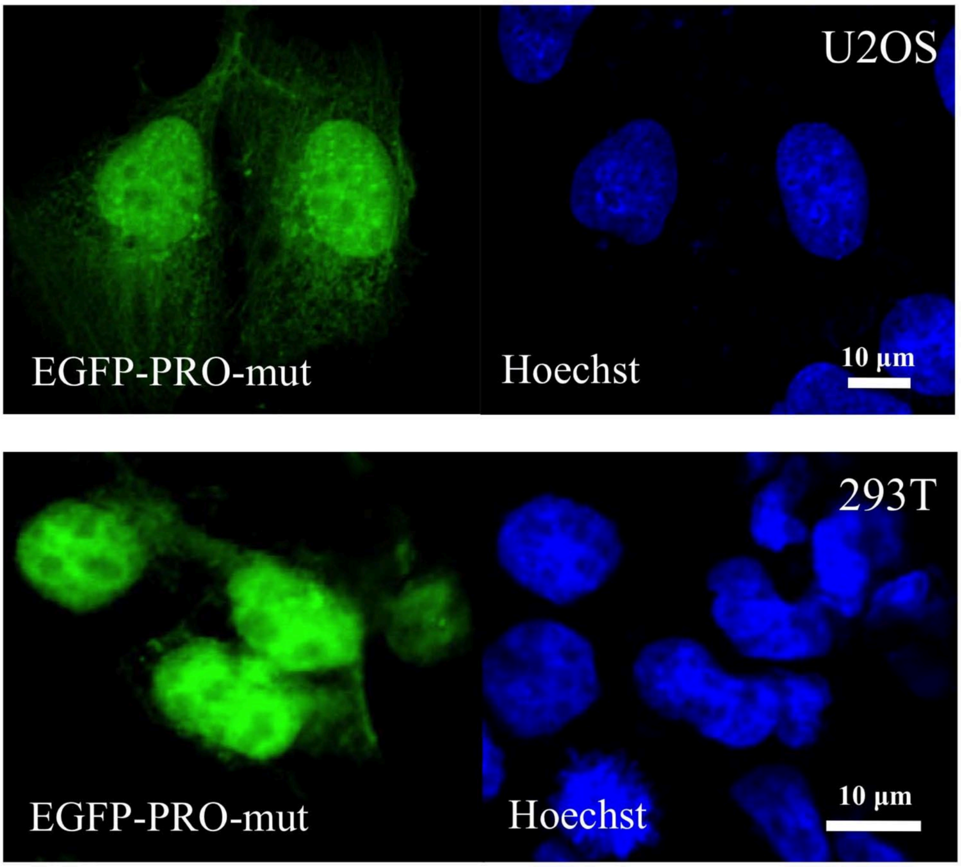

Figure S5. Localization of EGFP-Pro-mut in human osteosarcoma U2OS and HEK293T cells. Cells were transiently transfected with an expression plasmid encoding a mutant form of HERV-K(HML-2) Pro that is unable to self-process (see the main paper text for details). Cells were fixed on coverslips with 4% [w/v] paraformaldehyde and examined using a Nikon Eclipse Ti-A1 confocal microscope with NIS-Elements AR software. Fluorescence images were taken for EGFP and Hoechst 33342 counterstaining.

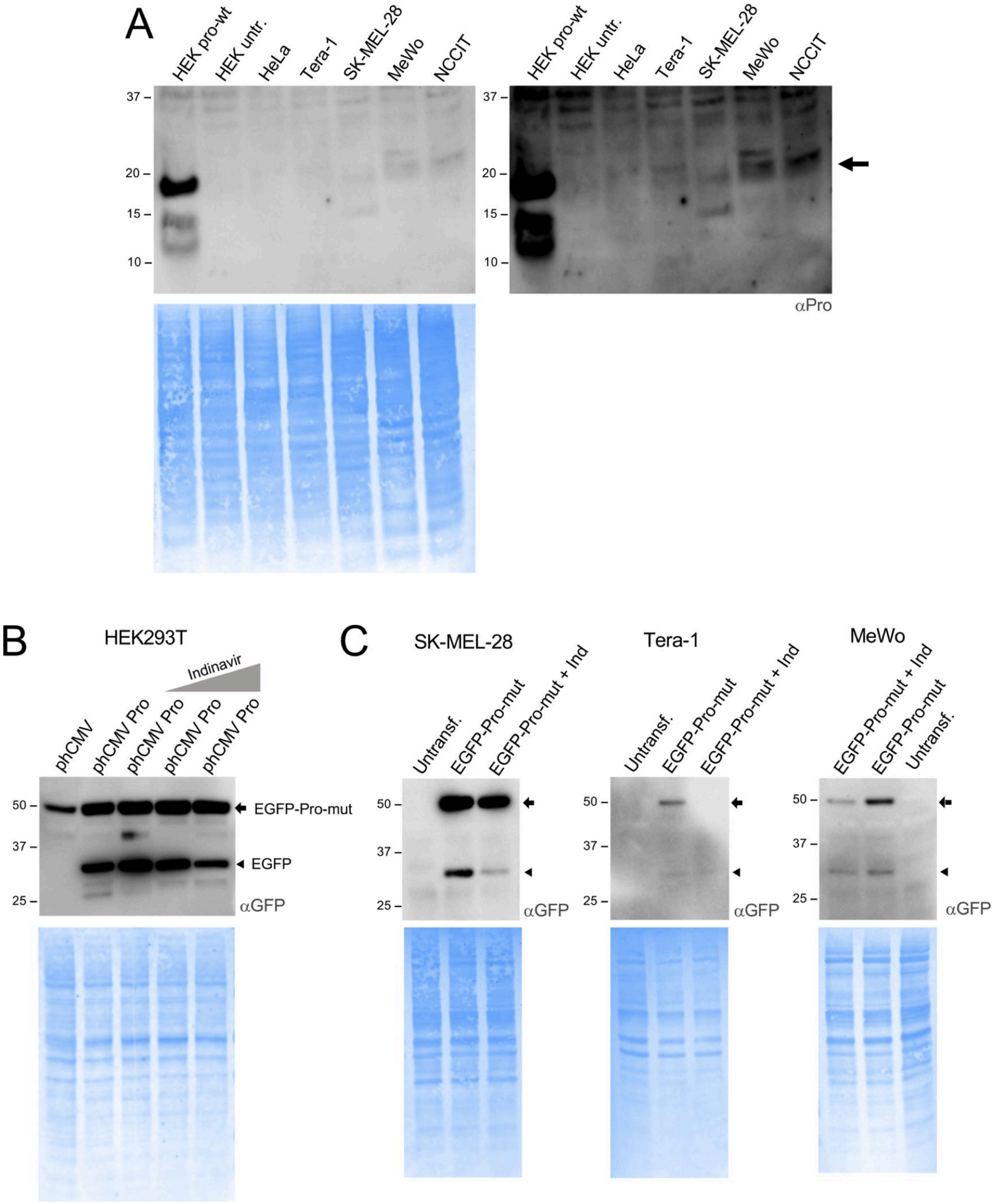
A
HEK pro-wt
HEK untr.
HeLa
Tera-1
SK-MEL-28
MeWo
NCCIT
37
20
15
10
αPro
B
HEK293T
Indinavir
phCMV
phCMV Pro
EGFP-Pro-mut
EGFP
αGFP
50
37
25
C
SK-MEL-28
Tera-1
MeWo
Untransf.
EGFP-Pro-mut
EGFP-Pro-mut + Ind

Figure S6

Figure S6. Evidence for presence of HERV-K(HML-2) Protease in cell lines known to express HERV-K(HML-2) at relatively high levels.

A. Evidence for presence of HERV-K(HML-2) Pro protein in cell lines known to overexpress HERV-K(HML-2) RNA and protein. Total protein lysates from cell lines Tera-1 and NCCIT, derived from testicular germ cell tumors (GCT), and melanoma-derived SK-MEL-28 and MeWo cell lines were subjected to SDS-PAGE and Western blotting followed by detection of HML-2 Pro using a polyclonal α-HML-2 Pro antibody (see the main paper text for details). Wild-type HML-2 Pro transiently expressed in HEK293T cells served as a positive control for self-processed HML-2 Pro, and untransfected HEK293T cells and HeLa cells served as negative controls. Note the protein bands detected by the α-Pro antibody in GCT and melanoma cell lines coinciding in size with the protein band detected in HEK293T cells expressing HML-2 wild-type Pro. A longer exposure time for the same blot membrane that better depicts protein bands of interest is shown on the right. The blot membrane stained with Coomassie Brilliant Blue after the ECL procedure is shown on the bottom left as a protein loading control. Note that different regions of the HML-2 Pro ORF are present, and thus were expressed, from plasmid constructs used for prokaryotic and eukaryotic expression of HML-2 Pro. In our experiments, the self-processed Pro migrated at approximately 18 kDa, a size coinciding with the precursor of the prokaryotically expressed HML-2 Pro. See the Methods section in the main paper text and furthermore compare with Fig. S1 and Fig. 6Aa, Ab, Ac). **B** and **C.** Evidence for presence of enzymatically active HML-2 Pro in cell lines known to overexpress HERV-K(HML-2). **B.** In a control experiment, HML-2 wild-type Pro (phCMV Pro) was co-expressed in HEK293T cells together with a fusion protein consisting of EGFP and mutant Pro (EGFP-Pro-mut), with that mutant Pro being unable to self-process (see Fig. 6 and Fig. S1). EGFP-Pro-mut (arrow) is processed in the presence of HML-2 wild-type Pro, as shown by the smaller-sized, "free" EGFP-portion (arrowhead) detected by an α-GFP antibody. The amount of "free" EGFP appears reduced in the presence of retroviral Protease inhibitor Indinavir. **C.** Transient expression of EGFP-Pro-mut in cell lines overexpressing HERV-K(HML-2), and likely HML-2 Pro (see **A**). No HML-2 wild-type Pro was co-expressed in this experiment. Note the presence of "free" EGFP (arrowhead) when EGFP-Pro-mut (arrow) is expressed indicating presence of enzymatically active HML-2 Pro in the respective cell lines. Also note that the amount of "free" EGFP was reduced in the presence of Indinavir, this being especially pronounced in the case of SK-MEL-28 cells. Sizes of marker proteins are indicated. Membranes were stained with Coomassie after the ECL step to document equal amounts of protein loaded.
